# Real-world effectiveness of early remdesivir in reducing mortality among vulnerable patients hospitalized for COVID-19: Evidence for clinical pharmacists and inpatient care providers

**DOI:** 10.1093/ajhp/zxag037

**Published:** 2026-02-11

**Authors:** Paul Loubet, Aastha Chandak, Susan Spivey, Yohei Doi, Alpesh N Amin, Neera Ahuja, Veronika Müller, Paul E Sax

**Affiliations:** Virulence Bactérienne et Infections Chroniques, INSERM U1047, Univ Montpellier, Service des Maladies Infectieuses et Tropicales, CHU Nîmes, Nîmes, France; Evidence and Access, Certara, Radnor, Pennsylvania, USA; Medical Affairs, Gilead Sciences, Foster City, California, USA; Department of Microbiology and Infectious Diseases, Fujita Health University School of Medicine, Toyoake, Japan; Division of Infectious Diseases, University of Pittsburgh School of Medicine, Pittsburgh, PA, USA; Department of Medicine, School of Medicine, University of California Irvine, Irvine, California, USA; Department of Internal Medicine, Stanford University School of Medicine, Stanford, CA, USA; Department of Pulmonology, Semmelweis University, Budapest, Hungary; Division of Infectious Diseases, Brigham and Women’s Hospital, Boston, Massachusetts, USA

**Keywords:** antiviral therapy, real-world data, remdesivir, SARS-CoV-2, stewardship

## Abstract

**Purpose:**

The aim of this study was to evaluate the effectiveness of remdesivir among vulnerable patients hospitalized with a primary diagnosis of coronavirus disease 2019 (COVID-19).

**Methods:**

In this retrospective study, data from the Premier Healthcare Database compiled from December 2021 to December 2024 were examined. Four cohorts were analyzed: overall (≥18 years of age), elderly (≥65 years of age), those with pneumonia due to COVID-19, and those with chronic obstructive pulmonary disease (COPD). Analyses were stratified by supplemental oxygen requirements upon admission. Patients treated with remdesivir within the first 2 days of hospitalization were matched to those not treated with remdesivir during hospitalization, using 1:1 propensity score matching without replacement. Outcomes of interest were 14- and 28-day all-cause inpatient mortality.

**Results:**

A total of 220,677 patients met the eligibility criteria; of these, 123,388 (55.9%) were treated with remdesivir within the first 2 days of hospitalization. Overall, treatment with remdesivir was associated with significantly lower 14- and 28-day mortality rates compared to rates in patients who did not receive remdesivir (adjusted hazard ratio [95% CI], 0.76 [0.73-0.79] and 0.78 [0.75-0.81], respectively; *P* < 0.0001). Similar results were observed across all patient groups irrespective of supplemental oxygen requirements and across early (December 2021-December 2022) and later (January 2023-December 2024) Omicron periods.

**Conclusions:**

These results build on previous research highlighting the effectiveness of early treatment initiation with remdesivir in vulnerable patients hospitalized due to SARS-CoV-2 infection.

Key Points

- The risk of severe outcomes associated with SARS-CoV-2 infection continues to be particularly significant for hospitalized vulnerable populations including the elderly, patients with pneumonia due to COVID-19, and those with chronic obstructive pulmonary disease.
- Evidence from this real-world study expands upon the previously established effectiveness of remdesivir in reducing inpatient mortality among vulnerable patient populations during the Omicron-predominant period.
- The phaseout of government-funded pandemic relief places clinical pharmacists at the forefront to bridge evidence-to-practice gaps and ensure improved survival outcomes, particularly for high-risk patient populations.

Despite the transition of coronavirus disease 2019 (COVID-19) from a pandemic to an endemic disease, severe acute respiratory syndrome coronavirus 2 (SARS-CoV-2) infection continues to remain a major public health concern.^1–4^ The current test positivity rate of 6.7% in the US (as of September 27, 2025) indicates ongoing viral circulation with the potential for severe public health consequences.^5^ It remains one of the top 15 causes of death in the United States, with crude mortality rate of 3.2 per 100,000 population between January 2025 and September 2025.^5^

The threat posed by SARS-CoV-2 infection remains especially acute among vulnerable populations, including the elderly, individuals with chronic respiratory conditions such as chronic obstructive pulmonary disease (COPD), and those with an underlying immunocompromising condition.^6–9^ Although the overall number of deaths annually has declined since the peak of the pandemic, the case fatality rate for COVID-19 remains high, especially among vulnerable patient groups.^10,11^ Individuals 65 years of age or older have up to 140 times higher risk of all-cause mortality than young adults, and those above 85 years of age have 340 times higher risk.^6^ Age remains the strongest risk factor for severe outcomes in those with SARS-CoV-2 infection.^6^ Similarly, individuals with underlying respiratory conditions are more susceptible to hospitalization and critical respiratory failure when infected with SARS-CoV-2.^6,12,13^

SARS-CoV-2 infections continue to pose a burden on healthcare systems, including persistent strain on hospital resources and rising healthcare costs.^14^ As of July 19, 2025, the overall rate of SARS-CoV-2–associated hospitalizations during the 2024-2025 season was 80.2 per 100,000.^15^ Notably, the use of antiviral treatments such as remdesivir has been associated with significant reductions in mortality and disease progression among hospitalized patients with risk factors for severe SARS-CoV-2 infection.^16^ As the first US Food and Drug Administration–approved antiviral for COVID-19, remdesivir has played a pivotal role in mitigating the severity of disease by reducing hospitalization duration and lowering mortality rates across different SARS-CoV-2 variants.^17–19^ Moreover, real-world studies have been foundational in not only confirming the initial evidence from randomized controlled trials (RCTs) but also in providing evidence on the effectiveness of remdesivir in the full spectrum of patients hospitalized for COVID-19, especially vulnerable patients.^7,8,18,20–23^

With the phaseout of government-funded pandemic relief drug acquisition programs and treatment guidelines, hospitals now need to integrate COVID-19 treatments, including remdesivir, into routine financial and operational planning, as is done for other essential therapies. This shift places additional financial pressure on institutions, increasing the demand for robust and up-to-date evidence to guide decisions on the use of antivirals like remdesivir, as pharmacy leaders balance competing therapeutic priorities and costs with the mandate to preserve optimal clinical outcomes, particularly for vulnerable patients. Using a geographically diverse hospital database in the United States, we aim to provide up-to-date evidence on the effectiveness of remdesivir in reducing inpatient mortality during the Omicron-predominant period among vulnerable patient populations including the elderly, those with pneumonia due to COVID-19, and those with COPD.

## Methods

### Study design and data source

This retrospective, comparative effectiveness study was conducted using patient-level data from the Premier Healthcare Database (PHD); https://premierinc.com/) captured between December 2021 and December 2024. The PHD is a geographically diverse, Health Insurance Portability and Accountability Act–compliant, all-payor hospital administrative billing database covering approximately 25% of all inpatient hospitalizations in the US. It captures information on patient demographics, hospital characteristics, procedures and medications administered, diagnoses at admission and discharge, discharge status, costs, and resource utilization.^24^ Less than 1% of patient records in this database have any missing information across all data elements captured.

### Study population

Eligible patients included adults hospitalized with a primary discharge diagnosis of COVID-19 (International Classification of Diseases, 10th Revision, Clinical Modification [ICD-10-CM] code U07.1) flagged as “presenton-admission.” Only the first COVID-19 hospitalization for a patient was included. Patients were excluded if they met any of the following criteria: pregnancy, incomplete data fields, transfer to or from another hospital, transfer from a hospice facility, admission for elective procedures, discharge or death during the first 2 days of hospitalization, use of extracorporeal membrane oxygenation at baseline, and initiation of remdesivir after the first 2 days of hospitalization. In addition, since some hospitals do not bill separately for supplemental oxygen supply or devices and instead include these charges in room charges, it may not be possible to identify supplemental oxygen use in such hospitals. Accordingly, only patients admitted to hospitals that reported separate charges for supplemental oxygen were included in the study.

### Study outcome

The outcome of interest was all-cause inpatient mortality at 14 and 28 days after the first 2 days of hospitalization, defined as a discharge status of “expired” or “hospice.” The baseline time of first 2 days was assessed equally in both groups, and patients who died or were discharged before day 3, ie, during the baseline period, were excluded to address immortal time bias. Patients were followed up starting on day 3 of hospitalization (after assessing remdesivir treatment status and baseline supplemental oxygen requirements) until death, discharge to hospice, or the end of follow-up. For patients discharged alive and not to a hospice setting, mortality outcomes were censored at 14 and 28 days after discharge.

A total of 4 patient cohorts were analyzed: ≥18 years old (the overall population), elderly patients (≥65 years, further divided into 65-74 years, 75-84 years, and ≥85 years; elderly population), those with pneumonia due to COVID-19 (ICD-10-CM code J12.82; pneumonia population), and those with COPD (ICD-10-CM code J43.x, J44.x; COPD population).

### Study variables

All baseline variables were examined within the first 2 days of hospitalization and included demographics, key comorbidities, hospital characteristics, hospital ward on admission, admission source, concomitant COVID-19 treatments at baseline, and baseline supplemental oxygen requirements. Since the actual time stamps are unavailable in the database, this definition of baseline provided all patients with a window of a minimum of 24 hours, during which clinical decisions were made and implemented (eg, for a patient admitted to a hospital at 11:59 PM, day 2 would start at midnight). Supplemental oxygenation status was determined as the highest level of support recorded in the billing records within the first 2 days of the hospitalization, ie, the same time period during which treatment with remdesivir was identified. Definitions for key study variables are described in supplementary eTable 1.

Treatment groups included those who received at least 1 dose of remdesivir within the first 2 days of admission (the remdesivir group) and those who did not receive remdesivir during hospitalization (the non-remdesivir group). Patients were categorized according to their baseline supplemental oxygen requirements (ie, within the first 2 days of hospitalization) as “no supplemental oxygen charges (NSOc)” and “any supplemental oxygen” charges. The supplemental oxygen charges were identified by the presence of any oxygen charges, including low-flow oxygen, high-flow oxygen/noninvasive ventilation, or invasive mechanical ventilation. Further information on these subgroups is available in previously published work.^7^

In addition, the overall study period was split into the early Omicron period (December 2021-December 2022) and later Omicron period (January 2023-December 2024), based on the predominant SARS-CoV-2 variants in the United States.^25,26^

### Statistical analysis

Propensity score (PS) methodology was used to balance the 2 treatment groups. To estimate the probability of receiving remdesivir, PSs were estimated using separate logistic regression models for the supplemental oxygen requirement categories to ensure valid comparability within the NSOc and any supplemental oxygen groups. In these models, exposure to remdesivir was the dependent variable and included baseline covariates (defined above); all covariates were retained in the model irrespective of their *P* value. Separate PSs were computed for each of the 4 patient cohorts analyzed in this study. Using the derived PSs, distribution of underlying confounders between the remdesivir and non-remdesivir groups was balanced using PS matching as the primary analysis. To account for differences in COVID-19 management practices across hospitals, patients were matched 1:1 without replacement, with a caliper distance set to 0.2 times the standard deviation of the logit of the PS. Patients were preferentially matched within the same hospital, followed by matching of unmatched patients within other hospitals of the same size that were using remdesivir. Patients were also matched within the same age group (18-49, 50-64, and ≥65 years) and admission month group (2- to 3-month blocks of admission month). Balance between the two groups was assessed at a threshold of absolute standardized mean difference (SMD) of <0.15.

The proportional hazard assumption was confirmed using a Kaplan-Meier curve and log of negative log plot. Cox proportional hazards models were used to assess 14- and 28-day in-hospital all-cause mortality, and adjusted hazard ratios (aHRs) and 95% confidence intervals (CIs) were derived. Details on the PS matching and Cox models employed in this study have been published previously.^7^ Sensitivity analysis was performed to assess the use of PS matching versus other balancing methods and to test the definition of the treatment groups. Two sensitivity analyses were performed in this study: (1) stabilized inverse probability of treatment weighting (IPTW) to PS matching,^27^ wherein the extreme PSs (<0.05 and >0.95) were trimmed; and (2) examining patients who received remdesivir within 2 days of admission versus those who did not receive remdesivir within 2 days of admission (the non-remdesivir group includes those patients initiated on remdesivir after the first 2 days).

This study was reviewed by the Advarra Institutional Review Board (Protocol #Pro00091215) and was determined to be exempt from IRB oversight in accordance with the Department of Health and Human Services regulations found at 45 CFR 46.104(d)(4).

## Results

**Figure 1.**
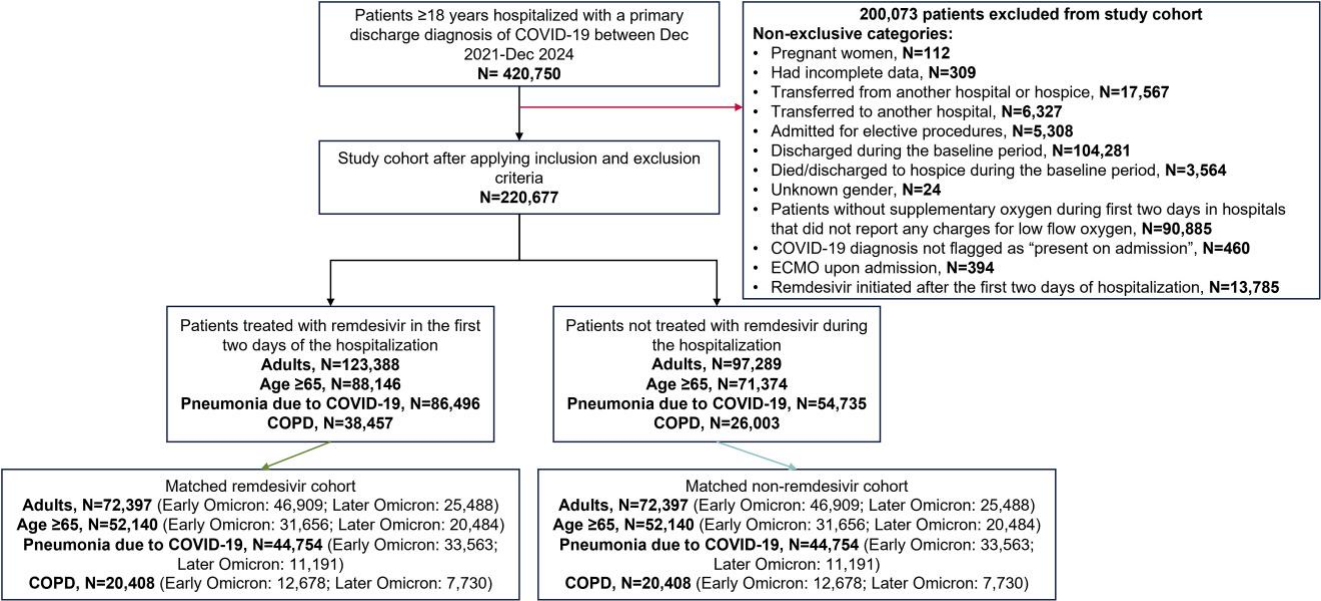
Study flow diagram. COVID-19 indicates coronavirus disease 2019; COPD, chronic obstructive pulmonary disease; ECMO, extracorporeal membrane oxygenation.

During the study period, 420,750 adult patients were hospitalized with a primary discharge diagnosis of COVID-19 and 220,677 patients met the eligibility criteria (Figure 1).

### Overall population

Of the eligible patients, 123,388 (55.9%) were treated with remdesivir within the first 2 days of hospitalization, while 97,289 (44.1%) did not receive remdesivir at any point during their hospitalization (Figure 1). Before PS matching, most of the patients in the remdesivir and non-remdesivir groups were 65 years of age or older (71.4% and 73.4%) and had cardiovascular disease (87.5% and 89.1%), respectively. More than one-third of patients had chronic pulmonary disease (39.5% and 34.1%), and about half of the patients required supplemental oxygen (55.7% and 44.4%). The median (interquartile range [IQR]) duration of remdesivir use was 5.0 (3.0-5.0) days. After matching, all baseline characteristics were well balanced, with an absolute SMD of <0.15 between the 2 groups (Table 1 and supplementary eFigure 1). Baseline demographics and clinical characteristics of patients hospitalized for COVID-19 before and after stabilized IPTW are described in supplementary eTable 2.

**Table 1.** Demographics of Patients Hospitalized for COVID-19 During the Period December 2021-December 2024 Before and After PS Matching (Overall Population)

Characteristic	Before PS matching			After PS matching		
	No remdesivir (n = 97,289)	Remdesivir (n = 123,388)	Absolute SMD	No remdesivir (n = 72,397)	Remdesivir (n = 72,397)	Absolute SMD
**Age group, years**						
18-49	8,161 (8.4)	10,576 (8.6)	0.07	5,237 (7.2)	5,237 (7.2)	0.00
50-64	17,754 (18.2)	24,666 (20.0)		13,414 (18.5)	13,414 (18.5)	
≥65	71,374 (73.4)	88,146 (71.4)		53,746 (74.2)	53,746 (74.2)	
**Female sex**	50,429 (51.8)	63,476 (51.4)	0.01	37,419 (51.7)	37,347 (51.6)	0.00
**Race**						
White	73,236 (75.3)	94,295 (76.4)	0.11	55,554 (76.7)	55,688 (76.9)	0.00
Black	15,510 (15.9)	16,281 (13.2)		10,287 (14.2)	10,138 (14.0)	
Asian	1,757 (1.8)	3,096 (2.5)		1,423 (2.0)	1,426 (2.0)	
Other	6,786 (7.0)	9,716 (7.9)		5,133 (7.1)	5,145 (7.1)	
**Ethnicity**						
Hispanic	7,655 (7.9)	13,045 (10.6)	0.11	5,985 (8.3)	5,834 (8.1)	0.00
Non-Hispanic	82,263 (84.6)	102,379 (83.0)		61,500 (84.9)	61,582 (85.1)	
Unknown	7,371 (7.6)	7,964 (6.5)		4,912 (6.8)	4,981 (6.9)	
**Primary payor**						
Commercial	11,684 (12.0)	17,768 (14.4)	0.08	9,182 (12.7)	9,073 (12.5)	0.00
Medicare	72,648 (74.7)	89,409 (72.5)		54,235 (74.9)	54,239 (74.9)	
Medicaid	7,879 (8.1)	10,456 (8.5)		5,484 (7.6)	5,522 (7.6)	
Other	5,078 (5.2)	5,755 (4.7)		3,496 (4.8)	3,563 (4.9)	
**Admission source**						
Transfer from SNF or ICF	3,645 (3.7)	5,013 (4.1)	0.02	2,857 (3.9)	2,825 (3.9)	0.00
**Hospital size, No. of beds**						
<100	7,292 (7.5)	8,964 (7.3)	0.13	5,536 (7.6)	5,436 (7.5)	0.03
100-199	16,355 (16.8)	20,879 (16.9)		12,036 (16.6)	12,136 (16.8)	
200-299	20,234 (20.8)	24,592 (19.9)		15,076 (20.8)	14,809 (20.5)	
300-399	19,001 (19.5)	21,551 (17.5)		13,632 (18.8)	13,545 (18.7)	
400-499	10,330 (10.6)	12,597 (10.2)		7,660 (10.6)	8,014 (11.1)	
≥500	24,077 (24.7)	34,805 (28.2)		18,457 (25.5)	18,457 (25.5)	
**Hospital location**						
Urban	84,637 (87.0)	109,129 (88.4)	0.04	63,203 (87.3)	63,277 (87.4)	0.00
Rural	12,652 (13.0)	14,259 (11.6)		9,194 (12.7)	9,120 (12.6)	
**Teaching hospital**	43,490 (44.7)	57,898 (46.9)	0.04	32,580 (45.0)	32,762 (45.3)	0.01
**Region**						
Midwest	24,007 (24.7)	28,681 (23.2)	0.19	18,086 (25.0)	17,763 (24.5)	0.00
Northeast	11,980 (12.3)	22,737 (18.4)		9,915 (13.7)	10,175 (14.1)	
South	50,844 (52.3)	56,674 (45.9)		35,986 (49.7)	36,128 (49.9)	
West	10,458 (10.7)	15,296 (12.4)		8,410 (11.6)	8,331 (11.5)	
**Comorbid conditions**						
Obesity	25,787 (26.5)	35,810 (29.0)	0.06	20,084 (27.7)	20,125 (27.8)	0.00
Chronic pulmonary disease	33,205 (34.1)	48,733 (39.5)	0.11	26,844 (37.1)	27,000 (37.3)	0.00
Cardiovascular disease	86,656 (89.1)	107,993 (87.5)	0.05	64,384 (88.9)	64,413 (89.0)	0.00
Diabetes mellitus	39,109 (40.2)	48,203 (39.1)	0.02	28,835 (39.8)	28,919 (39.9)	0.00
Renal disease	33,284 (34.2)	32,613 (26.4)	0.17	23,208 (32.1)	22,916 (31.7)	0.01
Cancer	6,603 (6.8)	9,625 (7.8)	0.04	5,234 (7.2)	5,297 (7.3)	0.00
**Immunocompromising condition**	15,780 (16.2)	22,430 (18.2)	0.05	12,459 (17.2)	12,473 (17.2)	0.00
**Hospital ward on admission**						
General ward	82,454 (84.8)	100,777 (81.7)	0.08	60,850 (84.1)	61,046 (84.3)	0.01
ICU/step-down unit	14,835 (15.2)	22,611 (18.3)		11,547 (15.9)	11,351 (15.7)	
**Key diagnosis on admission**						
Sepsis	481 (0.5)	506 (0.4)	0.01	336 (0.5)	349 (0.5)	0.00
Pneumonia	5,863 (6.0)	7,903 (6.4)	0.02	4,628 (6.4)	4,633 (6.4)	0.00
**Other COVID-19 treatments at baseline**						
Anticoagulants	70,460 (72.4)	97,298 (78.9)	0.15	54,886 (75.8)	54,932 (75.9)	0.00
Convalescent plasma	33 (0.0)	123 (0.1)	0.52	27 (0.0)	27 (0.0)	0.00
Corticosteroids	60,471 (62.2)	104,376 (84.6)	0.03	55,491 (76.6)	55,497 (76.7)	0.00
Baricitinib	4,753 (4.9)	5,933 (4.8)	0.07	3,958 (5.5)	3,938 (5.4)	0.00
Tocilizumab	2,090 (2.1)	3,989 (3.2)	0.00	1,895 (2.6)	1,933 (2.7)	0.00
Oral antivirals	2,449 (2.5)	376 (0.3)	0.19	303 (0.4)	279 (0.4)	0.01
**Baseline supplemental oxygen requirements**						
NSOc	54,089 (55.6)	54,657 (44.3)	0.25	36,361 (50.2)	36,361 (50.2)	0.00
LFO	26,571 (27.3)	40,962 (33.2)		22,498 (31.1)	22,498 (31.1)	
HFO/NIV	13,476 (13.9)	24,677 (20.0)		11,717 (16.2)	11,717 (16.2)	
IMV	3,153 (3.2)	3,092 (2.5)		1,821 (2.5)	1,821 (2.5)	
**Omicron period**						
Early (Dec 2021-Dec 2022)	62,836 (64.6)	77,039 (62.4)	0.04	46,909 (64.8)	46,909 (64.8)	0.00
Later (Jan 2023-Dec 2024)	34,453 (35.4)	46,349 (37.6)		25,488 (35.2)	25,488 (35.2)	

Abbreviations: COVID-19, coronavirus disease 2019; HFO/NIV, high-flow oxygen/noninvasive ventilation; ICF, intermediate care facility; ICU, intensive care unit; IMV, invasive mechanical ventilation; LFO, low-flow oxygen; NSOc, no supplemental oxygen charges; PS, propensity score; SMD, standardized mean difference; SNF, skilled nursing facility.

^a^Data are presented as No. (%).

**Figure 2.**
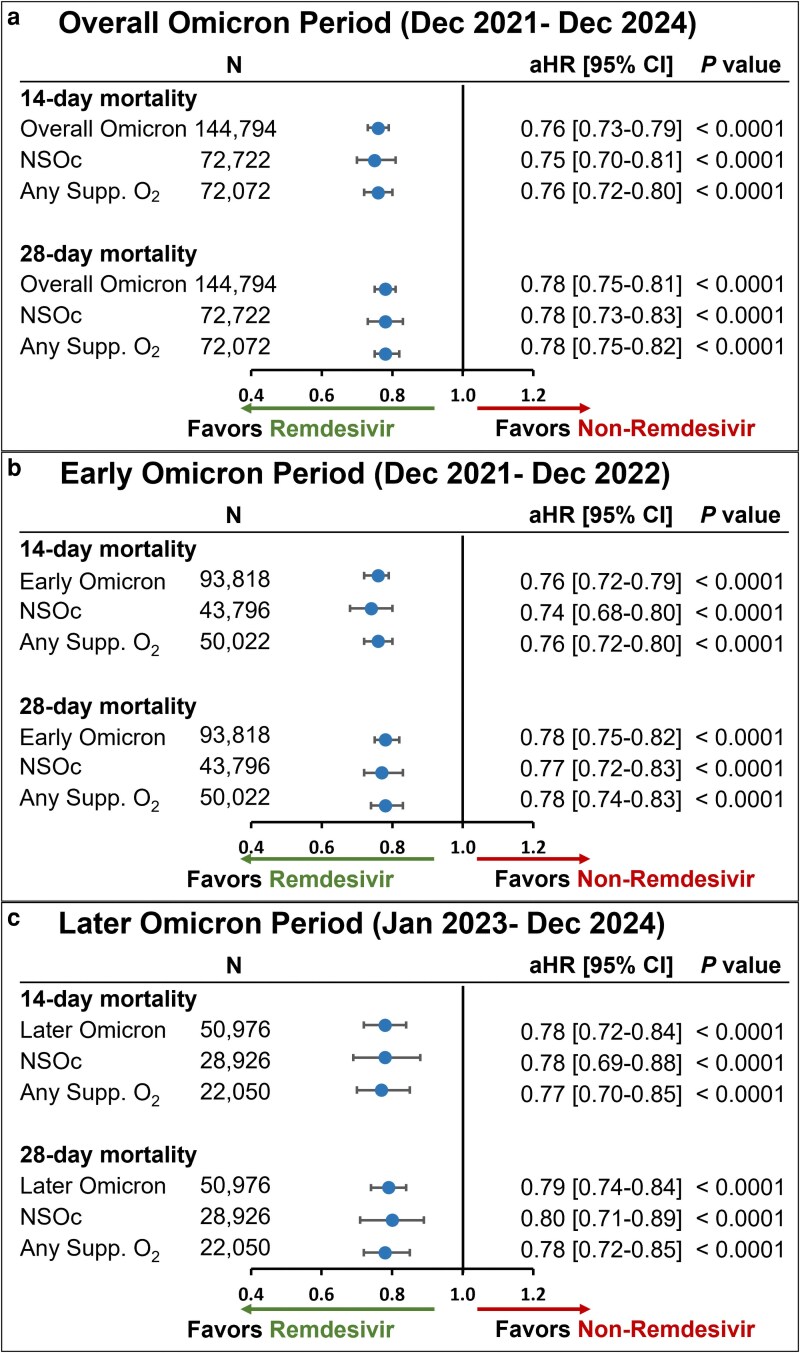
14- and 28-day mortality in patients hospitalized for coronavirus disease 2019 (COVID-19) treated with remdesivir within the first 2 days of hospitalization versus those not treated with remdesivir during hospitalization, by maximal supplemental oxygen requirements, after propensity score matching in the overall population. A Cox proportional hazards model was used to derive estimates adjusted for admission month and time-varying treatment with other COVID-19 medications (baricitinib, tocilizumab, and oral antivirals) during (a) the overall Omicron period, (b) the early Omicron period, and (c) the later Omicron period. aHR indicates adjusted hazard ratio; CI, confidence interval; NSOc, no supplemental oxygen charges; Supp. O_2_, supplemental oxygen.

The unadjusted all-cause inpatient mortality rate in the crude population prior to PS matching was consistently lower in the remdesivir group versus the non-remdesivir group in the overall Omicron group as well as subgroups of patients with NSOc and those who received any supplemental oxygen (supplementary eTable 3). Post PS matching, this mortality benefit in the remdesivir group remained consistent. In the PS-matched cohort, the unadjusted all-cause inpatient mortality rate at 14 days was 6.9% in the remdesivir group versus 8.8% in the non-remdesivir group, while at 28 days, the mortality rate was 9.1% versus 11.2%, respectively (supplementary eTable 4). After adjustment for baseline and clinical covariates, treatment with remdesivir resulted in significantly lower 14- and 28-day mortality rates compared to rates in patients who did not receive remdesivir (aHR [95% CI], 0.76 [0.73-0.79] and 0.78 [0.75-0.81], respectively) (*P* < 0.0001) (Figure 2a). Similar findings were observed across the early (Figure 2b) and later Omicron periods (Figure 2c).

In the NSOc subpopulation, unadjusted mortality risk was 4.2% versus 5.4% at 14 days and 5.5% versus 6.6% at 28 days for the remdesivir and non-remdesivir groups, respectively (supplementary eTable 4). After adjustment for baseline and clinical covariates, treatment with remdesivir resulted in significantly lower 14- and 28-day mortality rates compared to rates in patients who did not receive remdesivir (aHR [95% CI], 0.75 [0.70-0.81] and 0.78 [0.73-0.83], respectively) (*P* < 0.0001) (Figure 2a). Similar findings for early and later Omicron periods are shown in Figure 2b and 2c.

In the any supplemental oxygen subgroup, unadjusted mortality risk was 9.6% versus 12.2% at 14 days and 12.8% versus 15.8% at 28 days for the remdesivir and non-remdesivir groups, respectively (supplementary eTable 4). After adjustment for baseline and clinical covariates, treatment with remdesivir resulted in significantly lower 14- and 28-day mortality rates compared to rates in patients who did not receive remdesivir (aHR [95% CI], 0.76 [0.72-0.80] and 0.78 [0.75-0.82], respectively) (*P* < 0.0001) (Figure 2a). Similar findings for early and later Omicron periods are shown in Figure 2b and 2c.

These findings were also consistent in the sensitivity analyses conducted with IPTW (supplementary eTable 5) as well as in the sensitivity analysis using PS matching to compare remdesivir initiation versus no remdesivir initiation within the first 2 days of admission (supplementary eTable 6).

### Elderly patients with SARS-CoV-2 infection (elderly population)

Of the 159,520 elderly patients, 88,146 (55.3%) were treated with remdesivir within the first 2 days of hospitalization, while 71,374 (44.7%) did not receive remdesivir at any point during their hospitalization (Figure 1). Before PS matching, a higher proportion of patients in the remdesivir and non-remdesivir groups had COPD (40.3% and 34.7%) and required supplemental oxygen (53.4% and 42.8%), respectively. The median (IQR) duration of remdesivir use was 4.0 (3.0-5.0) days. After 1:1 matching, all baseline characteristics were well balanced, with an absolute SMD of <0.15 between the remdesivir and non-remdesivir groups (supplementary eTable 7 and supplementary eFigure 2). Baseline demographics and clinical characteristics of the elderly population before and after IPTW are described in supplementary eTable 8.

**Figure 3.**
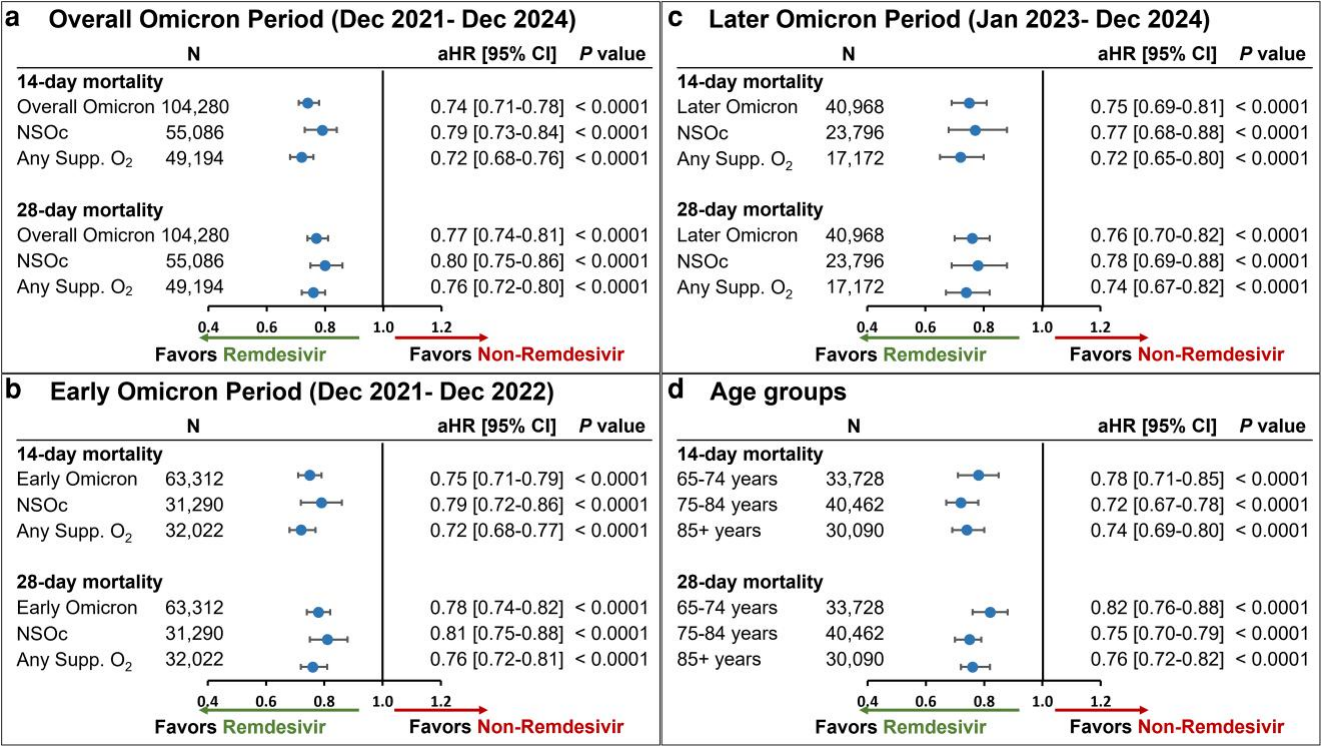
14- and 28-day mortality in patients hospitalized for coronavirus disease 2019 (COVID-19) treated with remdesivir within the first 2 days of hospitalization versus those not treated with remdesivir during hospitalization, by maximal supplemental oxygen requirements, after propensity score matching in the elderly population. A Cox proportional hazards model was used to derive estimates adjusted for admission month and time-varying treatment with other COVID-19 medications (baricitinib, tocilizumab, and oral antivirals) during (a) the overall Omicron period, (b) the early Omicron period, (c) the later Omicron period, and (d) by age group (65-74 years, 75-84 years, and ≥85 years). aHR indicates adjusted hazard ratio; CI, confidence interval; NSOc, no supplemental oxygen charges; Supp. O_2_, supplemental oxygen.

The unadjusted all-cause inpatient mortality rate in the crude population prior to PS matching was consistently lower in the remdesivir group versus non-remdesivir group in the overall Omicron group as well as the NSOc and any supplemental oxygen subgroups (supplementary eTable 3). Post PS matching, this mortality benefit in the remdesivir group remained consistent. In the PS-matched cohort, the unadjusted all-cause inpatient mortality rate at 14 days was 7.9% in the remdesivir group versus 10.3% in the non-remdesivir group, while at 28 days, the mortality rate was 10.2% versus 12.7%, respectively (supplementary eTable 9). After adjustment for baseline and clinical covariates, treatment with remdesivir resulted in significantly lower 14- and 28-day mortality rates compared to rates in patients who did not receive remdesivir (aHR [95% CI], 0.74 [0.71-0.78] and 0.77 [0.74-0.81], respectively) (*P* < 0.0001) (Figure 3a). These findings were consistent for the early (Figure 3b) and later Omicron periods (Figure 3c) and across age groups (65-74 years, 75-84 years, and ≥85 years) (Figure 3d).

In the NSOc subpopulation PS-matched cohort, the unadjusted mortality risk was 5.3% versus 6.5% at 14 days and 6.6% versus 7.8% at 28 days for the remdesivir and non-remdesivir groups, respectively (supplementary eTable 9). After adjustment for baseline and clinical covariates, treatment with remdesivir resulted in significantly lower 14- and 28-day mortality rates compared to rates in patients who did not receive remdesivir (aHR [95% CI], 0.79 [0.73-0.84] and 0.80 [0.75-0.86], respectively) (*P* < 0.0001) (Figure 3a). Similar findings for early and later Omicron periods are shown in Figure 3b and 3c.

In the any supplemental oxygen subgroup PS-matched cohort, the unadjusted mortality risk was 10.8% versus 14.5% at 14 days and 14.2% versus 18.1% at 28 days for the remdesivir and non-remdesivir groups, respectively (supplementary eTable 9). After adjustment for baseline and clinical covariates, treatment with remdesivir resulted in significantly lower 14- and 28-day mortality rates compared to rates in patients who did not receive remdesivir (aHR [95% CI], 0.72 [0.68-0.76] and 0.76 [0.72-0.80], respectively) (*P* < 0.0001) (Figure 3a). Additional data for early and later Omicron periods are shown in Figure 3b and 3c, respectively.

These findings were also consistent in the sensitivity analyses conducted with IPTW (supplementary eTable 10) as well as in the sensitivity analysis using PS matching to compare remdesivir initiation versus no remdesivir initiation within the first 2 days of admission (supplementary eTable 11).

### Patients with SARS-CoV-2 infection and pneumonia due to COVID-19 (pneumonia population)

Of the 141,231 patients in the pneumonia population, 86,496 (61.2%) were treated with remdesivir within the first 2 days of hospitalization, while 54,735 (38.8%) did not receive remdesivir during their hospitalization (Figure 1). Before PS matching, most of the patients in the remdesivir and non-remdesivir groups were 65 years of age or older (68.2% and 70.8%, respectively) and had cardiovascular disease (86.4% and 88.9%), and most patients required supplemental oxygen (62.5% and 57.3%). The median (IQR) duration of remdesivir use was 5.0 (4.0-5.0) days. After 1:1 matching, all baseline characteristics were well balanced, with an absolute SMD of <0.15 between the remdesivir and non-remdesivir groups (supplementary eTable 12 and supplementary eFigure 3). A majority of the patients (∼60%) received supplemental oxygen (supplementary eTable 12). Baseline demographics and clinical characteristics of the pneumonia population before and after IPTW are described in supplementary eTable 13.

**Figure 4.**
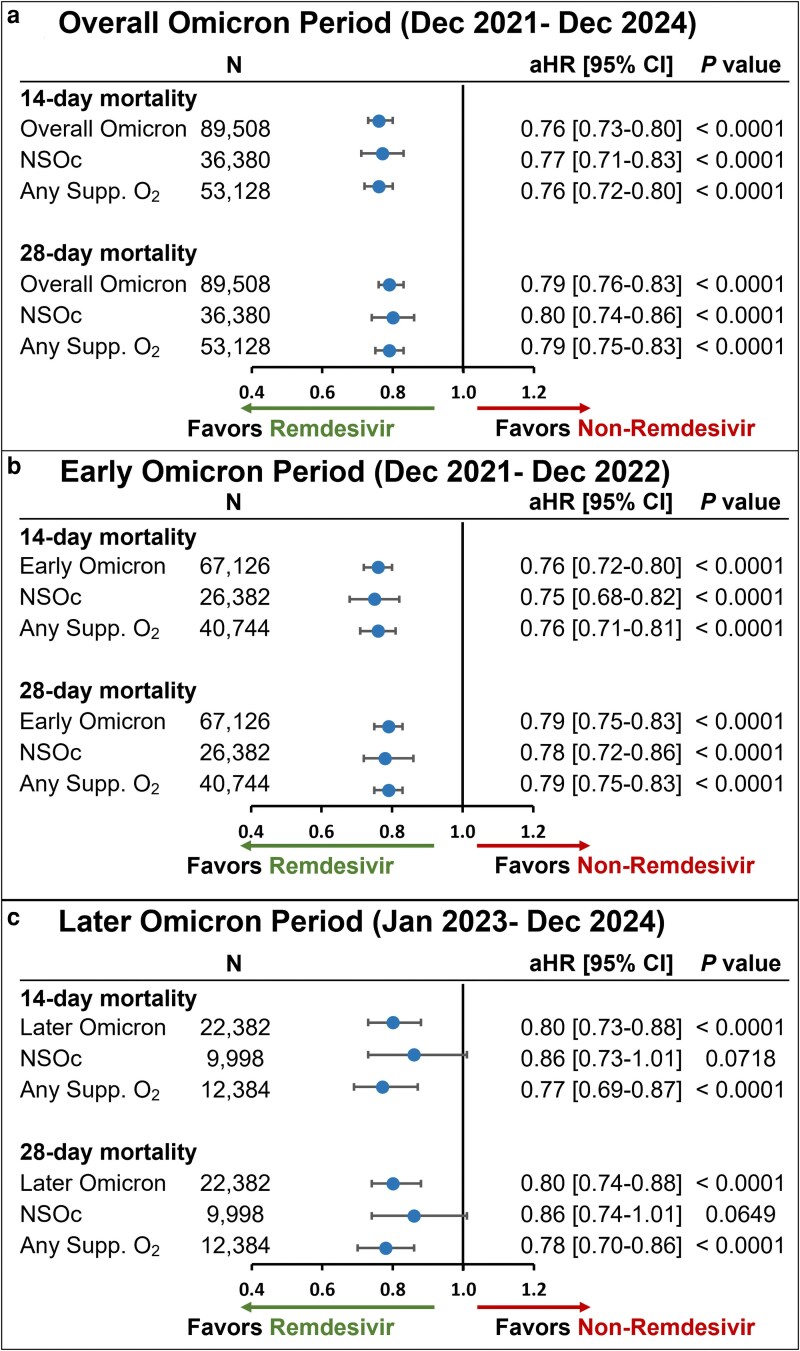
14- and 28-day mortality in patients hospitalized for coronavirus disease 2019 (COVID-19) treated with remdesivir within the first 2 days of hospitalization versus those not treated with remdesivir during hospitalization, by maximal supplemental oxygen requirements, after propensity score matching in the pneumonia population. A Cox proportional hazards model was used to derive estimates adjusted for admission month and time-varying treatment with other COVID-19 medications (baricitinib, tocilizumab, and oral antivirals) during (a) the overall Omicron period, (b) the early Omicron period, and (c) the later Omicron period. aHR indicates adjusted hazard ratio; CI, confidence interval; NSOc, no supplemental oxygen charges; Supp. O_2_, supplemental oxygen.

The unadjusted all-cause inpatient mortality rate in the crude population prior to PS matching was consistently lower in the remdesivir group versus the non-remdesivir group in the overall Omicron group as well as the NSOc and any supplemental oxygen subgroups (supplementary eTable 3). Post PS matching, this mortality benefit in the remdesivir group remained consistent. In the PS-matched cohort, the unadjusted all-cause inpatient mortality rate at 14 days was 9.1% in the remdesivir group versus 11.5% in the non-remdesivir group, while at 28 days, the mortality rate was 12.3% versus 15.0%, respectively (supplementary eTable 14). After adjustment for baseline and clinical covariates, treatment with remdesivir resulted in significantly lower 14- and 28-day mortality rates compared to rates in patients who did not receive remdesivir (aHR [95% CI], 0.76 [0.73-0.80] and 0.79 [0.76-0.83], respectively) (*P* < 0.0001) (Figure 4a). These findings were consistent for the early (Figure 4b) and later Omicron periods (Figure 4c).

In the NSOc subpopulation PS-matched cohort, unadjusted mortality risk for the entire Omicron period was 6.0% versus 7.5% at 14 days and 8.0% versus 9.5% at 28 days for the remdesivir and non-remdesivir groups, respectively (supplementary eTable 14). After adjustment for baseline and clinical covariates, treatment with remdesivir resulted in significantly lower 14- and 28-day mortality rates compared to rates in patients who did not receive remdesivir (aHR [95% CI], 0.77 [0.71-0.83] and 0.80 [0.74-0.86], respectively) (*P* < 0.0001) (Figure 4a). Similar findings were observed across the early (Figure 4b) and later Omicron periods (Figure 4c).

In the any supplemental oxygen subgroup PS-matched cohort, unadjusted mortality risk for the entire Omicron period was 11.1% versus 14.2% at 14 days and 15.2% versus 18.7% at 28 days for the remdesivir and non-remdesivir groups, respectively (supplementary eTable 14). After adjustment for baseline and clinical covariates, treatment with remdesivir resulted in significantly lower 14- and 28-day mortality rates compared to rates in patients who did not receive remdesivir (aHR [95% CI], 0.76 [0.72-0.80] and 0.79 [0.75-0.83], respectively) (*P* < 0.0001) (Figure 4a). Similar findings for the early and later Omicron periods are shown in Figure 4b and 4c, respectively.

These findings were also consistent in the sensitivity analyses conducted with IPTW (supplementary eTable 15) as well as in the sensitivity analysis using PS matching to compare remdesivir initiation versus no remdesivir initiation within the first 2 days of admission (supplementary eTable 16).

### Patients with SARS-CoV-2 Infection and COPD (COPD Population)

Of the 64,460 patients with SARS-CoV-2 infection and COPD, 38,457 (59.7%) were treated with remdesivir within the first 2 days of hospitalization, while 26,003 (40.3%) did not receive remdesivir at any point during their hospitalization (Figure 1). Before PS matching, most of the patients in the remdesivir and non-remdesivir groups were 65 years of age or older (77.9% and 79.3%, respectively), had cardiovascular disease (92.6% and 94.0%), and required supplemental oxygen (66.9% and 60.5%). The median (IQR) duration of remdesivir use was 5.0 (3.0-5.0) days. After 1:1 matching, all baseline characteristics were well balanced, with an absolute SMD of <0.15 between the remdesivir and non-remdesivir groups (supplementary eTable 17 and supplementary eFigure 4). A majority of the patients (∼63%) received supplemental oxygen (supplementary eTable 17). Baseline demographics and clinical characteristics of the COPD population before and after IPTW are described in supplementary eTable 18.

**Figure 5.**
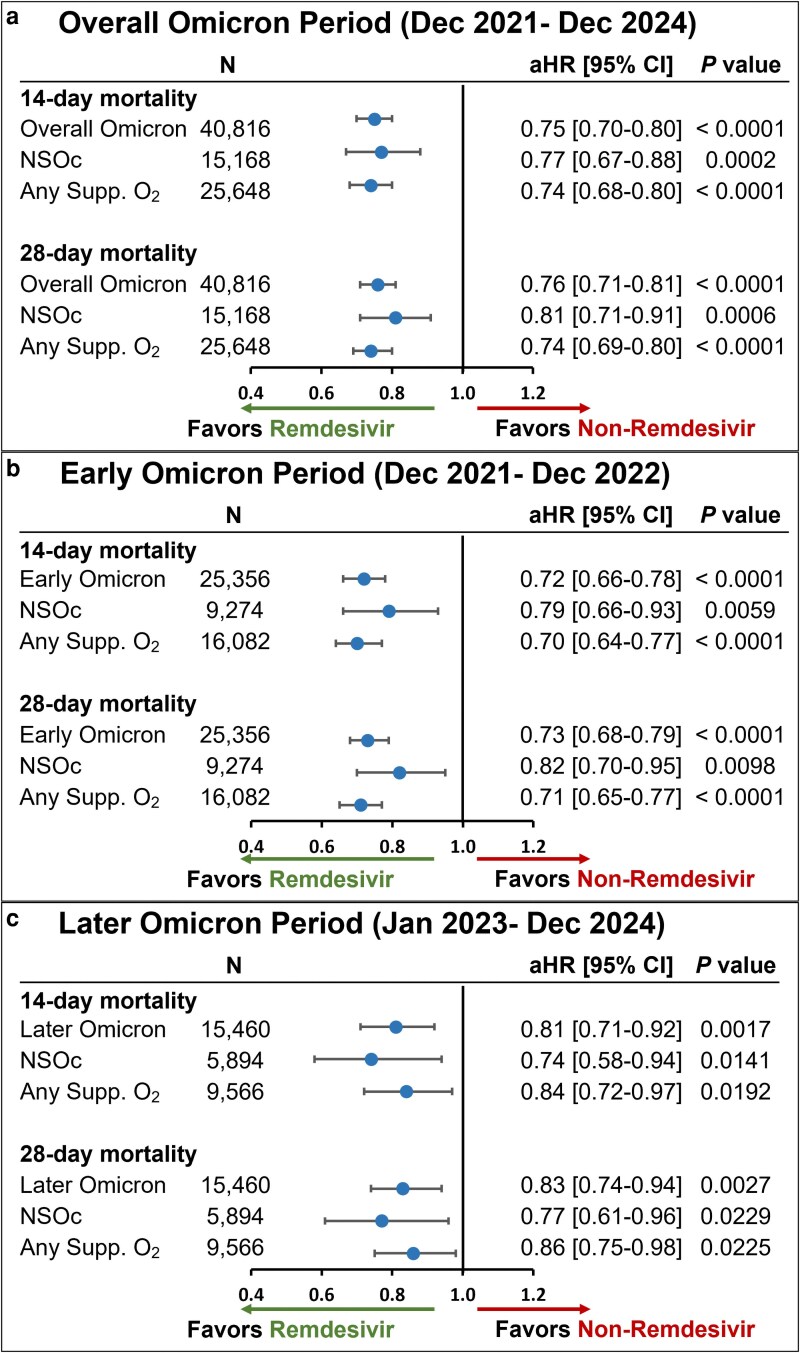
14- and 28-day mortality in patients hospitalized for coronavirus disease 2019 (COVID-19) treated with remdesivir within the first 2 days of hospitalization versus those not treated with remdesivir during hospitalization, by maximal supplemental oxygen requirements, after propensity score matching in the population with chronic obstructive pulmonary disease. A Cox proportional hazards model was used to derive estimates adjusted for admission month and time-varying treatment with other COVID-19 medications (baricitinib, tocilizumab, and oral antivirals) during (a) the overall Omicron period, (b) the early Omicron period, and (c) the later Omicron period. aHR indicates adjusted hazard ratio; CI, confidence interval; NSOc, no supplemental oxygen charges; Supp. O_2_, supplemental oxygen.

The unadjusted all-cause inpatient mortality rate in the crude population prior to PS matching was consistently lower in the remdesivir group versus the non-remdesivir group in the overall Omicron group as well as subgroups of NSOc and any supplemental oxygen (supplementary eTable 3). Post PS matching, this mortality benefit in the remdesivir group remained consistent. In the PS-matched cohort, the unadjusted all-cause inpatient mortality rate at 14 days was 7.6% in the remdesivir group versus 9.9% in the non-remdesivir group, while at 28 days, the mortality rate was 9.7% and 12.4%, respectively (supplementary eTable 19). After adjustment for baseline and clinical covariates, treatment with remdesivir resulted in significantly lower 14- and 28-day mortality rates compared to rates in patients who did not receive remdesivir (aHR [95% CI], 0.75 [0.70-0.80] and 0.76 [0.71-0.81], respectively) (*P* < 0.0001) (Figure 5a). These findings were consistent for the early (Figure 5b) and later Omicron periods (Figure 5c).

In the NSOc subpopulation PS-matched cohort, unadjusted mortality risk for the entire Omicron period was 4.9% versus 6.1% at 14 days and 6.3% versus 7.4% at 28 days for the remdesivir and non-remdesivir groups, respectively (supplementary eTable 19). After adjustment for baseline and clinical covariates, treatment with remdesivir resulted in significantly lower 14- and 28-day mortality rates compared to rates in patients who did not receive remdesivir (aHR [95% CI], 0.77 [0.67-0.88] and 0.81 [0.71-0.91], respectively) (*P* < 0.0001) (Figure 5c). Similar findings for the early and later Omicron periods are shown in Figure 5b and 5c.

In the any supplemental oxygen subgroup PS-matched cohort, unadjusted mortality risk for the entire Omicron period was 9.2% versus 12.1% at 14 days and 11.7% versus 15.3% at 28 days for the remdesivir and non-remdesivir groups, respectively (supplementary eTable 19). After adjustment for baseline and clinical covariates, treatment with remdesivir resulted in significantly lower 14- and 28-day mortality rates compared to rates in patients who did not receive remdesivir (aHR [95% CI], 0.74 [0.68-0.80] and 0.74 [0.69-0.80], respectively) (*P* < 0.0001) (Figure 5a). Similar findings for the early and later Omicron periods are shown in Figure 5b and 5c.

These findings were also consistent in the sensitivity analyses conducted with IPTW (supplementary eTable 20) as well as in the sensitivity analysis using PS matching to compare remdesivir initiation versus no remdesivir initiation within the first 2 days of admission (supplementary eTable 21).

## Discussion

Management of patients hospitalized for SARS-CoV-2 infection has evolved substantially since the onset of the COVID-19 pandemic. However, SARS-CoV-2 infection remains a persistent clinical and public health challenge due to ongoing viral mutations, disparities in vaccinations, and waning immunity.^2,3^ As a result, vulnerable patients remain at a high risk for severe outcomes.^6,9^ In this study, treatment with remdesivir was associated with a statistically significant reduction in all-cause inpatient mortality at 14 and 28 days across all patient subgroups evaluated, independent of baseline disease severity as determined by supplemental oxygen requirement. Notably, this study provides new evidence supporting the clinical benefit of remdesivir among patients with SARS-CoV-2 infection and COPD, a patient subgroup that is at an increased risk for severe clinical outcomes.^6,12,13^ These findings are particularly valuable as clinical practice continues to shift toward a more nuanced, patient-centered model driven by real-world data and clinical judgment of multidisciplinary teams and stewardship strategies within hospitals. Furthermore, as budget pressures persist and grow across healthcare systems, these data provide quantification of the clinical benefits associated with the use of remdesivir so that these benefits can be considered in the context of the costs incurred.

Recognizing the evolving nature of SARS-CoV-2 and the growing perception that as mortality rates have declined in comparison to the pandemic era, there may be a reduced perceived urgency to consider broad use of a treatment such as remdesivir for patients hospitalized with SARS-CoV-2 infection. This analysis directly addresses this perception, with robust evidence that the case fatality rate for hospitalized COVID-19 patients remains persistently high, and that the effectiveness of remdesivir in reducing this rate is substantial during the later Omicron-dominant period as it has been across the pandemic and into the endemic era. The clinical benefit associated with remdesivir remained consistent across these periods, suggesting that its antiviral efficacy is preserved across evolving SARS-CoV-2 strains and across variations in disease severity. Consistent with findings from previous studies,^7,8,19,23,28–32^ results from this study reinforce the effectiveness of remdesivir in vulnerable patient populations, particularly as SARS-CoV-2 continues to evolve in the endemic phase.

Despite the well-established efficacy and safety profile of remdesivir and recommendations for its routine use in patients hospitalized for SARS-CoV-2 infection by major treatment guidelines such as those of the National Institutes of Health (NIH), Infectious Diseases Society of America, and World Health Organization (WHO),^18,21,23,33–36^ a significant proportion of patients do not receive remdesivir. In this study, approximately 44% of high-risk patients eligible to receive remdesivir did not receive remdesivir upon admission (Table 1). These patients experienced 22% higher mortality at 28 days compared to those who were treated with remdesivir, highlighting a critical gap in the implementation of evidence-based therapies and guideline recommendations. This finding aligns with the results from RCTs and real-world studies, which have consistently demonstrated that early remdesivir administration is associated with reduced mortality, decreased progression to invasive mechanical ventilation, and shorter hospital stays.^7,8,18,19,21,23,28–32,37^ The observed underutilization of remdesivir in clinical practice may reflect barriers such as delayed diagnosis, clinical uncertainty regarding patient eligibility, or institutional variation in treatment protocols or order sets. These insights underscore the need for up-to-date evidence to ensure timely and equitable access to remdesivir, particularly for vulnerable populations at a high risk for severe outcomes.

While treatment guidelines are available for managing patients hospitalized due to SARS-CoV-2 infection,^33–35^ the majority of them are predominantly informed by RCTs conducted during the early phase of the pandemic when host immune response was a hallmark of SARS-CoV-2 infection.^36^ With the shift in the presentation of SARS-CoV-2 infection, treatment with an antiviral has assumed greater clinical importance. Moreover, the pivotal trials were often underpowered to detect treatment effects within vulnerable patient subgroups such as elderly patients, immunocompromised individuals, and those with comorbidities. In addition, the recent retirement of the NIH COVID-19 treatment guidelines that integrated evidence from both RCTs and real-world studies may introduce further uncertainty in therapeutic decision-making.^34^ In this evolving landscape, clinical pharmacists not only continue to be an essential part of the inpatient care team but also leverage their expertise in interpreting the latest evidence to bridge evidence-to-practice gaps and proactively identifying patients who may benefit from treatment with an antiviral such as remdesivir. Moreover, providing the pharmacy community with current, real-world data on patient outcomes and treatment effectiveness is essential to inform stewardship, treatment decisions, order sets, and hospital protocols.

One of the key strengths of this study is leveraging one of the most comprehensive electronic healthcare data repositories in the US, covering approximately 25% of all hospitalizations, thus offering a highly representative view of real-world clinical practice. As such, the findings not only reflect the patterns of clinical practice in the general population but also help to identify critical gaps in the implementation of evidence-based treatments,^24^ particularly in vulnerable patient populations. To address potential bias inherent in observational studies, we employed a rigorous PS matching and multivariable adjustment to account for measurable baseline differences between treatment groups. Furthermore, detailed subgroup analysis allowed for evaluation of clinical outcomes in various subgroups of a vulnerable population who have been underrepresented or excluded from earlier RCTs. Moreover, we utilized IPTW, a method that retains all patients in the cohort and does not exclude any unmatched individuals, and the results remained consistent.

While the methodology employed in this study has notable strengths, several limitations must be acknowledged. As detailed in the previous publication,^7^ the retrospective design of the study introduces the possibility for residual confounding despite control for known prognostic variables. Misclassification bias may also occur as key clinical variables such as comorbid conditions, treatments, and procedures are derived from administrative data including billing and ICD-10 codes, which may underreport or inaccurately capture certain diagnoses or treatments. Finally, a lack of long-term follow-up beyond hospital discharge can lead to incomplete outcome assessment, introduce potential bias, and limit the generalizability of findings. Despite these limitations, the use of a large, diverse, and contemporary patient population, combined with rigorous analytical methods, provides meaningful insight into the continued clinical value of remdesivir in vulnerable patient populations in the endemic phase of SARS-CoV-2.

## Conclusion

Findings from this study reinforce the clinical benefit of remdesivir in reducing all-cause inpatient mortality rates among vulnerable patients, including the elderly, those with a secondary diagnosis of pneumonia due to COVID-19, or those with COPD. The persistently high mortality observed in the later Omicron period, combined with the consistently demonstrated clinical effectiveness of remdesivir across different SARS-CoV-2 variants and disease severity, underscores its broad applicability in the management of patients hospitalized due to SARS-CoV-2 infection. Active engagement of clinical pharmacists is crucial to translating these findings into practice and ensuring improved survival outcomes for high-risk patient populations.

## Supplementary Material

zxag037_Supplementary_Data

## Data Availability

The data supporting this study’s findings are available from Premier, Inc. (https://www.premierinc.com/). Restrictions apply to the availability of these data, which were used under license for this study.

## Additional information

The authors provided the following information regarding their contributions to this work: All authors contributed substantially to the manuscript and agreed to the final submitted version.
