# Supplementary material for: Real-world effectiveness of early remdesivir in reducing mortality among vulnerable patients hospitalized for COVID-19: Evidence for clinical pharmacists and inpatient care providers
Source: Am J Health Syst Pharm. 2026 Feb 11;83(Suppl 3):S2915–30. doi: 10.1093/ajhp/zxag037 (PMC13070694; doi:10.1093/ajhp/zxag037)
Supplement: zxag037_Supplementary_Data [file zxag037_supplementary_data.docx]

**eTable 1. Definitions of Key Study Variables**

| Key Study Variables | | Definitions |
| --- | --- | --- |
| Remdesivir treatment |  | Billing charges for treatment: Remdesivir  ICD-10 procedure codes: XW033E5, XW043E5 |
| Key comorbidities | Obesity | ICD-10-CM diagnosis codes: E66, Z6825-Z6845 |
|  | Chronic pulmonary disease | ICD-10-CM diagnosis codes: I27.8, I27.9, J40, J41.x, J42, J43.x, J44.x, J45.x, J46, J47.x, J60, J61, J62.x, J63.x, J64, J65, J66.x, J67.x, J68.4, J70.1, J70.3 |
|  | Cardiovascular disease (including hypertension) | ICD-10-CM diagnosis codes: I00-I99 |
|  | Diabetes Mellitus | ICD-10-CM diagnosis codes: E10-E14 |
|  | Renal disease | ICD-10-CM diagnosis codes: I12.0, I13.1, N03.2, N03.3, N03.4, N03.5, N03.6, N03.7, N05.2, N05.3, N05.4, N05.5, N05.6, N05.7, N18.x, N19.x, N25.0, Z49.0, Z49.1, Z49.2, Z94.0, Z99.2 |
|  | Cancer | ICD-10-CM diagnosis codes: C00-C96 |
|  |  |  |
| Immunocompromising condition |  | ICD-10-CM code for cancer (C00-C96), transplant (Z94.x), hematologic malignancies (C81.x, C82.x, C83.x, C84.x, C85.x, C88.x, C90.x, C91.x, C92.x, C93.x, C94.x, C95.x, C96.x), primary immunodeficiencies (D80.x, D81.x, D82.x, D83.x, D84.x, G11.3, E70.330, D71.x, D70.x), asplenia (Q89.01, Z90.81), toxic effects of antineoplastics (T45.1x), bone marrow failure/aplastic anemia (D61.x), severe combined immunodeficiencies (D80.x, D81.x, D82.x, D83.x, D84.x, D86.x, D89.0, D89.1, D89.2, D89.3, D89.4x, D89.81, D89.82, D89.89, D89.9), HIV (B20), patients with chronic graft-versus-host disease or who are taking immunosuppressive medications for another indication (Z89.8x, Z79.52, Z79.61, Z79.62x, Z79.63x, Z79.64, Z79.69, Z79.810, Z79.811, Z79.818) |
| Supplemental oxygen requirements | IMV | Billing charges for devices: invasive mechanical ventilation, tracheostomy, endotracheal tube intubation |
|  | ECMO | Billing charges for devices: extracorporeal membrane oxygenation |
|  | HFO/NIV | Billing charges for devices: negative-pressure ventilation, positive-pressure ventilation, CPAP, BiPAP, high-flow system via nasal cannula, venturi face mask, rebreather, non-rebreather mask, positive expiratory pressure |
|  | LFO | Billing charges for devices/oxygen supply: simple face mask, oxygen pendant, low-flow system via nasal cannula, oxygen supply |
|  | NSOc | No billing charges for IMV, ECMO, HFO/NIV, or LFO at baseline |
| Admitting diagnosis | Sepsis | ICD-10-CM diagnosis codes: A02.1, A32.7, A40.x, A41.x, A42.7, A54.86, B37.7, R65.20, R65.21, T81.44X |
|  | Pneumonia | ICD-10-CM diagnosis codes: J12.x, J13, J14, J15.x, J16.x, J17, J18.x, A48.1, B25.0, A37.01, A37.11, A37.81, A37.91, A22.1, B44.0, B77.81, J10.00, J10.01, J10.08, J11.00, J11.08 |
| Baseline medications | Anticoagulants | Billing charges for treatment at baseline: apixaban, argatroban, desirudin, lepirudin, dabigatran, danaparoid, edoxaban, tinzaparin, heparin (excluding use of heparin flush), ardeparin, bivalirudin |
|  | Corticosteroids | Billing charges for treatment at baseline: prednisone, prednisolone, methylprednisolone, hydrocortisone, dexamethasone |
|  | Convalescent plasma | Billing charges for treatment at baseline: convalescent plasma; ICD-10 procedure codes: XW13325, XW14325 |
|  | Tocilizumab | Billing charges for treatment at baseline: tocilizumab; ICD-10 procedure codes: XW033H5, XW043H5 |
|  | Baricitinib | Billing charges for treatment at baseline: baricitinib; ICD-10 procedure codes: XW0DXM6, XW0H7M6, XW0G7M6 |
|  | Oral antivirals | Billing charges for treatment at baseline: nirmatrelvir ritonavir, molnupiravir |
| Abbreviations: BiPAP, bilevel positive airway pressure; CPAP, continuous positive airway pressure; COPD, chronic obstructive pulmonary disease; HFO/NIV, high-flow oxygen/non-invasive ventilation; ICD-10-CM, International Classification of Diseases, 10th Revision (Clinical Modification); LFO, low-flow oxygen; IMV, invasive mechanical ventilation; ECMO, extracorporeal membrane oxygenation; NSOc, no supplemental oxygen charges. | | |

**eTable 2. Demographics of Patients Hospitalized for COVID-19 During December 2021-December 2024 Before and After IPTW (Overall Population)**

| Characteristic | | Before IPTW | | | After IPTW | | |
| --- | --- | --- | --- | --- | --- | --- | --- |
|  |  | **No Remdesivir**  **n = 97,289** | **Remdesivir**  **n = 123,388** | **Absolute SMD** | **No Remdesivir** | **Remdesivir** | **Absolute SMD** |
| Age group, years | 18–49 | 8,161 (8.4) | 10,576 (8.6) | 0.07 | 8.4 | 8.4 | 0.00 |
|  | 50–64 | 17,754 (18.2) | 24,666 (20.0) |  | 19.3 | 19.2 |  |
|  | ≥65 | 71,374 (73.4) | 88,146 (71.4) |  | 72.3 | 72.4 |  |
| Gender | Female | 50,429 (51.8) | 63,476 (51.4) | 0.01 | 51.5 | 51.5 | 0.00 |
| Race | White | 73,236 (75.3) | 94,295 (76.4) | 0.11 | 76.0 | 76.0 | 0.00 |
|  | Black | 15,510 (15.9) | 16,281 (13.2) |  | 14.3 | 14.3 |  |
|  | Asian | 1,757 (1.8) | 3,096 (2.5) |  | 2.2 | 2.2 |  |
|  | Other | 6,786 (7.0) | 9,716 (7.9) |  | 7.5 | 7.5 |  |
| Ethnicity | Hispanic | 7,655 (7.9) | 13,045 (10.6) | 0.11 | 9.4 | 9.4 | 0.00 |
|  | Non-Hispanic | 82,263 (84.6) | 102,379 (83.0) |  | 83.8 | 83.8 |  |
|  | Unknown | 7,371 (7.6) | 7,964 (6.5) |  | 6.8 | 6.8 |  |
| Primary payor | Commercial | 11,684 (12.0) | 17,768 (14.4) | 0.08 | 13.3 | 13.3 | 0.04 |
|  | Medicare | 72,648 (74.7) | 89,409 (72.5) |  | 73.5 | 73.6 |  |
|  | Medicaid | 7,879 (8.1) | 10,456 (8.5) |  | 8.3 | 8.2 |  |
|  | Other | 5,078 (5.2) | 5,755 (4.7) |  | 4.9 | 4.9 |  |
| Admission source | Transfer from SNF or ICF | 3,645 (3.7) | 5,013 (4.1) | 0.02 | 4.0 | 3.9 | 0.00 |
| Hospital size, number of beds | <100 | 7,292 (7.5) | 8,964 (7.3) | 0.13 | 7.6 | 7.5 | 0.04 |
|  | 100–199 | 16,355 (16.8) | 20,879 (16.9) |  | 16.7 | 16.8 |  |
|  | 200–299 | 20,234 (20.8) | 24,592 (19.9) |  | 20.1 | 20.4 |  |
|  | 300–399 | 19,001 (19.5) | 21,551 (17.5) |  | 18.0 | 18.0 |  |
|  | 400–499 | 10,330 (10.6) | 12,597 (10.2) |  | 10.7 | 10.6 |  |
|  | 500+ | 24,077 (24.7) | 34,805 (28.2) |  | 26.9 | 26.8 |  |
| Hospital location | Urban | 84,637 (87.0) | 109,129 (88.4) | 0.04 | 87.7 | 87.9 | 0.01 |
|  | Rural | 12,652 (13.0) | 14,259 (11.6) |  | 12.3 | 12.1 |  |
| Teaching hospital | | 43,490 (44.7) | 57,898 (46.9) | 0.04 | 46.1 | 46.1 | 0.00 |
| Region | Midwest | 24,007 (24.7) | 28,681 (23.2) | 0.19 | 24.1 | 24.2 | 0.00 |
|  | Northeast | 11,980 (12.3) | 22,737 (18.4) |  | 15.9 | 15.8 |  |
|  | South | 50,844 (52.3) | 56,674 (45.9) |  | 48.2 | 48.2 |  |
|  | West | 10,458 (10.7) | 15,296 (12.4) |  | 11.8 | 11.9 |  |
| Comorbid conditions | Obesity | 25,787 (26.5) | 35,810 (29.0) | 0.06 | 28.0 | 28.0 | 0.00 |
|  | Chronic pulmonary disease | 33,205 (34.1) | 48,733 (39.5) | 0.11 | 37.5 | 37.5 | 0.00 |
|  | Cardiovascular disease | 86,656 (89.1) | 10,7993 (87.5) | 0.05 | 88.3 | 88.3 | 0.00 |
|  | Diabetes Mellitus | 39,109 (40.2) | 48,203 (39.1) | 0.02 | 39.7 | 39.7 | 0.00 |
|  | Renal disease | 33,284 (34.2) | 32,613 (26.4) | 0.17 | 30.1 | 30.2 | 0.00 |
|  | Cancer | 6,603 (6.8) | 9,625 (7.8) | 0.04 | 7.5 | 7.4 | 0.00 |
| Immunocompromising condition | | 15,780 (16.2) | 22,430 (18.2) | 0.05 | 17.6 | 17.6 | 0.00 |
| Hospital ward on admission | General ward | 82,454 (84.8) | 100,777 (81.7) | 0.08 | 83.0 | 82.9 | 0.00 |
|  | ICU/step down unit | 14,835 (15.2) | 22,611 (18.3) |  | 17.0 | 17.1 |  |
| Key diagnosis on admission | Sepsis | 481 (0.5) | 506 (0.4) | 0.01 | 0.5 | 0.5 | 0.00 |
|  | Pneumonia | 5,863 (6.0) | 7,903 (6.4) | 0.02 | 6.2 | 6.3 | 0.00 |
| Other COVID-19 treatments at baseline | Anticoagulants | 70,460 (72.4) | 97,298 (78.9) | 0.15 | 76.1 | 75.9 | 0.00 |
|  | Convalescent plasma | 33 (0.0) | 123 (0.1) | 0.52 | 0.1 | 0.1 | 0.00 |
|  | Corticosteroids | 60,471 (62.2) | 104,376 (84.6) | 0.03 | 75.0 | 75.1 | 0.00 |
|  | Baricitinib | 4,753 (4.9) | 5,933 (4.8) | 0.07 | 5.0 | 5.0 | 0.00 |
|  | Tocilizumab | 2,090 (2.1) | 3,989 (3.2) | 0.00 | 2.9 | 2.8 | 0.00 |
|  | Oral antivirals | 2,449 (2.5) | 376 (0.3) | 0.19 | 1.1 | 1.1 | 0.00 |
| Baseline supplemental oxygen requirements | NSOc | 54,089 (55.6) | 54,657 (44.3) | 0.25 | 48.9 | 48.8 | 0.03 |
|  | LFO | 26,571 (27.3) | 40,962 (33.2) |  | 30.5 | 30.8 |  |
|  | HFO/NIV | 13,476 (13.9) | 24,677 (20.0) |  | 17.6 | 17.5 |  |
|  | IMV | 3,153 (3.2) | 3,092 (2.5) |  | 2.9 | 2.9 |  |
| Omicron period | Early (Dec 2021-Dec 2022) | 62,836 (64.6) | 77,039 (62.4) | 0.04 | 63.0 | 63.1 | 0.00 |
|  | Later (Jan 2023-Feb 2024) | 34,453 (35.4) | 46,349 (37.6) |  | 37.0 | 36.9 |  |
| Data is presented as n (%) before matching and as % after matching, unless otherwise indicated.  Abbreviations: COVID-19, coronavirus disease 2019; HFO/NIV, high-flow oxygen/non-invasive ventilation; ICF, intermediate care facility; ICU, intensive care unit; IMV, invasive mechanical ventilation; IPTW, inverse probability of treatment weighting; LFO, low-flow oxygen; NSOc, no supplemental oxygen charges; SMD, standardized mean difference; SNF, skilled nursing facility | | | | | | | |

**eTable 3. Unadjusted All-cause Inpatient Mortality Rates in the Overall Omicron Period (Crude Population prior to PS Matching)**

|  | **Overall Omicron** | | **NSOc** | | **Any Supplemental Oxygen** | |
| --- | --- | --- | --- | --- | --- | --- |
|  | **No Remdesivir** | **Remdesivir** | **No Remdesivir** | **Remdesivir** | **No Remdesivir** | **Remdesivir** |
| **Overall population** | **n=97289** | **n=123388** | **n=54089** | **n=54657** | **n=43200** | **n=68731** |
| 14-day crude mortality rate | 8272 (8.5) | 8324 (6.7) | 2874 (5.3) | 2285 (4.2) | 5398 (12.5) | 6039 (8.8) |
| 28-day crude mortality rate | 10408 (10.7) | 10999 (8.9) | 3501 (6.5) | 2933 (5.4) | 6907 (16.0) | 8066 (11.7) |
| **Elderly Population** | **n=71374** | **n=88146** | **n=40795** | **n=41104** | **n=30579** | **n=47042** |
| 14-day crude mortality rate | 7225 (10.1) | 7111 (8.1) | 2669 (6.5) | 2099 (5.1) | 4556 (14.9) | 5012 (10.7) |
| 28-day crude mortality rate | 8837 (12.4) | 9064 (10.3) | 3195 (7.8) | 2619 (6.4) | 5642 (18.5) | 6445 (13.7) |
| **Pneumonia Population** | **n=54735** | **n=86496** | **n=23373** | **n=32398** | **n=31362** | **n=54098** |
| 14-day crude mortality rate | 6325 (11.6) | 7181 (8.3) | 1749 (7.5) | 1755 (5.4) | 4576 (14.6) | 5426 (10.0) |
| 28-day crude mortality rate | 8206 (15.0) | 9672 (11.2) | 2225 (9.5) | 2312 (7.1) | 5981 (19.1) | 7360 (13.6) |
| **COPD Population** | **n=26003** | **n=38457** | **n=10269** | **n=12735** | **n=15734** | **n=25722** |
| 14-day crude mortality rate | 2499 (9.6) | 2739 (7.1) | 605 (5.9) | 576 (4.5) | 1894 (12.0) | 2163 (8.4) |
| 28-day crude mortality rate | 3124 (12.0) | 3495 (9.1) | 739 (7.2) | 735 (5.8) | 2385 (15.2) | 2760 (10.7) |

Data is presented as n (%), unless otherwise indicated.

Abbreviations: COPD, chronic obstructive pulmonary disease; PS, propensity score; NSOc, no supplemental oxygen charges.

**eTable 4. Unadjusted All-cause Inpatient Mortality Rates (PS matching without replacement, Overall Population)**

|  | **Early Omicron (Dec 2021 - Dec 2022)** | | **Later Omicron (Jan 2023- Dec 2024)** | | **Overall (Dec 2021- Dec 2024)** | |
| --- | --- | --- | --- | --- | --- | --- |
|  | **No Remdesivir during the hospitalization** | **Remdesivir in the first two days** | **No Remdesivir during the hospitalization** | **Remdesivir in the first two days** | **No Remdesivir during the hospitalization** | **Remdesivir in the first two days** |
| **Overall Omicron** | **n=46909** | **n=46909** | **n=25488** | **n=25488** | **n=72397** | **n=72397** |
| 14-day mortality | 4734 (10.1) | 3709 (7.9) | 1634 (6.4) | 1280 (5.0) | 6368 (8.8) | 4989 (6.9) |
| 28-day mortality | 6182 (13.2) | 5074 (10.8) | 1903 (7.5) | 1515 (5.9) | 8085 (11.2) | 6589 (9.1) |
| **NSOc** | **n=21898** | **n=21898** | **n=14463** | **n=14463** | **n=36361** | **n=36361** |
| 14-day mortality | 1317 (6.0) | 1037 (4.7) | 639 (4.4) | 502 (3.5) | 1956 (5.4) | 1539 (4.2) |
| 28-day mortality | 1672 (7.6) | 1405 (6.4) | 726 (5.0) | 581 (4.0) | 2398 (6.6) | 1986 (5.5) |
| **Any supplemental oxygen** | **n=25011** | **n=25011** | **n=11025** | **n=11025** | **n=36036** | **n=36036** |
| 14-day mortality | 3417 (13.7) | 2672 (10.7) | 995 (9.0) | 778 (7.1) | 4412 (12.2) | 3450 (9.6) |
| 28-day mortality | 4510 (18.0) | 3669 (14.7) | 1177 (10.7) | 934 (8.5) | 5687 (15.8) | 4603 (12.8) |

Data is presented as n (%), unless otherwise indicated.

Abbreviations: PS, propensity score; NSOc, no supplemental oxygen charges.

**eTable 5: 14- and 28-day Mortality in Patients Hospitalized for COVID-19 Treated with Remdesivir Within the First 2 Days of Hospitalization vs Those Not Treated with Remdesivir During Hospitalization, by Maximal Supplemental Oxygen Requirements (IPTW, Overall Population)**

|  | **aHR [95% CI]** | ***P* value** |
| --- | --- | --- |
| **14-day mortality** |  |  |
| Overall Omicron | 0.74 [0.71- 0.77] | < 0.0001 |
| NSOc | 0.74 [0.69-0.79] | < 0.0001 |
| Any supplemental oxygen | 0.75 [0.71-0.78] | < 0.0001 |
| **28-day mortality** |  |  |
| Overall Omicron | 0.77 [0.74-0.80] | < 0.0001 |
| NSOc | 0.76 [0.72-0.81] | < 0.0001 |
| Any supplemental oxygen | 0.78 [0.74-0.81] | < 0.0001 |

Cox proportional hazards model was used to derive estimates adjusted for admission month, and time-varying treatment with other COVID-19 medications (baricitinib, tocilizumab, oral antivirals).

Abbreviations: aHR, adjusted hazard ratio; CI, confidence interval; COVID-19, coronavirus disease 2019; IPTW, inverse probability of treatment weighting; NSOc, no supplemental oxygen charges.

**eTable 6. 14- and 28-day Mortality in Patients Hospitalized for COVID-19 Treated with Remdesivir Within the First 2 Days of Hospitalization vs Those Not Treated with Remdesivir Within the First 2 Days, by Maximal Supplemental Oxygen Requirements (PS matching, Overall Population)**

|  | **N** | **aHR [95% CI]** | ***P* value** |
| --- | --- | --- | --- |
| **14-day mortality** |  |  |  |
| Overall Omicron | 155,486 | 0.74 [0.71-0.77] | < 0.0001 |
| NSOc | 77,344 | 0.74 [0.69-0.79] | < 0.0001 |
| Any supplemental oxygen | 78,142 | 0.73 [0.70-0.77] | < 0.0001 |
| **28-day mortality** |  |  |  |
| Overall Omicron | 155,486 | 0.75 [0.72-0.78] | < 0.0001 |
| NSOc | 77,344 | 0.75 [0.70-0.79] | < 0.0001 |
| Any supplemental oxygen | 78,142 | 0.75 [0.72-0.79] | < 0.0001 |

Cox proportional hazards model was used to derive estimates adjusted for admission month, and time-varying treatment with other COVID-19 medications (baricitinib, tocilizumab, oral antivirals).

Abbreviations: aHR, adjusted hazard ratio; CI, confidence interval; COVID-19, coronavirus disease 2019; NSOc, no supplemental oxygen charges; PS, propensity score.

**eTable 7. Demographics of Patients Hospitalized for COVID-19 During December 2021-December 2024 Before and After PS matching (Elderly Population)**

| Characteristic | | Before PS matching | | | After PS matching | | |
| --- | --- | --- | --- | --- | --- | --- | --- |
|  |  | **No Remdesivir**  **n = 71,374** | **Remdesivir**  **n = 88,146** | **Absolute SMD** | **No Remdesivir**  **n = 52,140** | **Remdesivir**  **n = 52,140** | **Absolute SMD** |
| Age group, years | 65–74 | 22,351 (31.3) | 29,041 (32.9) | 0.04 | 16,864 (32.3) | 16,864 (32.3) | 0.00 |
|  | 75–84 | 27,619 (38.7) | 33,904 (38.5) |  | 20,231 (38.8) | 20,231 (38.8) |  |
|  | ≥85 | 21,404 (30.0) | 25,201 (28.6) |  | 15,045 (28.9) | 15,045 (28.9) |  |
| Gender | Female | 37,495 (52.5) | 46,035 (52.2) | 0.01 | 27,303 (52.4) | 27,328 (52.4) | 0.00 |
| Race | White | 56,402 (79.0) | 70,755 (80.3) | 0.12 | 41,843 (80.3) | 41,821 (80.2) | 0.05 |
|  | Black | 9,251 (13.0) | 9,148 (10.4) |  | 6,032 (11.6) | 5,910 (11.3) |  |
|  | Asian | 1,367 (1.9) | 2,440 (2.8) |  | 1,069 (2.1) | 1,113 (2.1) |  |
|  | Other | 4,354 (6.1) | 5,803 (6.6) |  | 3,196 (6.1) | 3,296 (6.3) |  |
| Ethnicity | Hispanic | 4,661 (6.5) | 8,107 (9.2) | 0.07 | 3,669 (7.0) | 3,642 (7.0) | 0.00 |
|  | Non-Hispanic | 61,297 (85.9) | 74,392 (84.4) |  | 44,930 (86.2) | 44,934 (86.2) |  |
|  | Unknown | 5,416 (7.6) | 5,647 (6.4) |  | 3,541 (6.8) | 3,564 (6.8) |  |
| Primary payor | Commercial | 2,702 (3.8) | 3,980 (4.5) | 0.11 | 2,181 (4.2) | 2,094 (4.0) | 0.00 |
|  | Medicare | 65,471 (91.7) | 80,380 (91.2) |  | 47,742 (91.6) | 47,781 (91.6) |  |
|  | Medicaid | 928 (1.3) | 1,356 (1.5) |  | 688 (1.3) | 694 (1.3) |  |
|  | Other | 2,273 (3.2) | 2,430 (2.8) |  | 1,529 (2.9) | 1,571 (3.0) |  |
| Admission source | Transfer from SNF or ICF | 3,279 (4.6) | 4,509 (5.1) | 0.02 | 2,487 (4.8) | 2,512 (4.8) | 0.00 |
| Hospital size, number of beds | <100 | 5,616 (7.9) | 6,406 (7.3) | 0.12 | 4,050 (7.8) | 4,026 (7.7) | 0.00 |
|  | 100–199 | 12,233 (17.1) | 14,891 (16.9) |  | 8,764 (16.8) | 8,788 (16.9) |  |
|  | 200–299 | 15,077 (21.1) | 17,624 (20.0) |  | 10,983 (21.1) | 10,745 (20.6) |  |
|  | 300–399 | 14,158 (19.8) | 15,489 (17.6) |  | 9,972 (19.1) | 9,963 (19.1) |  |
|  | 400–499 | 7,601 (10.6) | 9,149 (10.4) |  | 5,600 (10.7) | 5,847 (11.2) |  |
|  | 500+ | 16,689 (23.4) | 24,587 (27.9) |  | 12,771 (24.5) | 12,771 (24.5) |  |
| Hospital location | Urban | 61,766 (86.5) | 78,173 (88.7) | 0.07 | 45,498 (87.3) | 45,594 (87.4) | 0.01 |
|  | Rural | 9,608 (13.5) | 9,973 (11.3) |  | 6,642 (12.7) | 6,546 (12.6) |  |
| Teaching hospital | | 30,931 (43.3) | 41,207 (46.7) | 0.07 | 22,920 (44.0) | 23,215 (44.5) | 0.01 |
| Region | Midwest | 17,657 (24.7) | 20,114 (22.8) | 0.19 | 12,930 (24.8) | 12,622 (24.2) | 0.05 |
|  | Northeast | 9,065 (12.7) | 16,920 (19.2) |  | 7,452 (14.3) | 7,643 (14.7) |  |
|  | South | 37,032 (51.9) | 40,130 (45.5) |  | 25,800 (49.5) | 25,824 (49.5) |  |
|  | West | 7,620 (10.7) | 10,982 (12.5) |  | 5,958 (11.4) | 6,051 (11.6) |  |
| Comorbid conditions | Obesity | 15,576 (21.8) | 19,880 (22.6) | 0.02 | 11,722 (22.5) | 11,662 (22.4) | 0.00 |
|  | Chronic pulmonary disease | 24,762 (34.7) | 35,565 (40.3) | 0.12 | 19,717 (37.8) | 19,788 (38.0) | 0.00 |
|  | Cardiovascular disease | 67,078 (94.0) | 82,167 (93.2) | 0.03 | 48,815 (93.6) | 48,821 (93.6) | 0.00 |
|  | Diabetes Mellitus | 29,032 (40.7) | 34,592 (39.2) | 0.03 | 20,805 (39.9) | 20,863 (40.0) | 0.00 |
|  | Renal disease | 27,204 (38.1) | 26,926 (30.5) | 0.16 | 18,500 (35.5) | 18,361 (35.2) | 0.01 |
|  | Cancer | 5,361 (7.5) | 7,687 (8.7) | 0.04 | 4,120 (7.9) | 4,181 (8.0) | 0.00 |
| Immunocompromising condition | | 11,642 (16.3) | 16,335 (18.5) | 0.06 | 9,023 (17.3) | 9,011 (17.3) | 0.00 |
| Hospital ward on admission | General ward | 61,495 (86.2) | 72,640 (82.4) | 0.10 | 44,537 (85.4) | 44,702 (85.7) | 0.01 |
|  | ICU/step down unit | 9,879 (13.8) | 15,506 (17.6) |  | 7,603 (14.6) | 7,438 (14.3) |  |
| Key diagnosis on admission | Sepsis | 346 (0.5) | 368 (0.4) | 0.01 | 239 (0.5) | 243 (0.5) | 0.00 |
|  | Pneumonia | 4,208 (5.9) | 5,492 (6.2) | 0.01 | 3,194 (6.1) | 3,234 (6.2) | 0.00 |
| Other COVID-19 treatments at baseline | Anticoagulants | 51,355 (72.0) | 68,179 (77.3) | 0.12 | 39,065 (74.9) | 39,071 (74.9) | 0.00 |
|  | Convalescent plasma | 19 (0.0) | 58 (0.1) | 0.51 | 17 (0.0) | 13 (0.0) | 0.00 |
|  | Corticosteroids | 43,066 (60.3) | 72,889 (82.7) | 0.02 | 38,954 (74.7) | 38,975 (74.8) | 0.00 |
|  | Baricitinib | 2,763 (3.9) | 3,043 (3.5) | 0.05 | 2,118 (4.1) | 2,104 (4.0) | 0.00 |
|  | Tocilizumab | 1,172 (1.6) | 2,057 (2.3) | 0.02 | 1,017 (2.0) | 1,032 (2.0) | 0.00 |
|  | Oral antivirals | 2,040 (2.9) | 314 (0.4) | 0.20 | 222 (0.4) | 218 (0.4) | 0.00 |
| Baseline supplemental oxygen requirements | NSOc | 40,795 (57.2) | 41,104 (46.6) | 0.21 | 27,543 (52.8) | 27,543 (52.8) | 0.00 |
|  | LFO | 19,736 (27.7) | 29,156 (33.1) |  | 16,197 (31.1) | 16,197 (31.1) |  |
|  | HFO/NIV | 9,051 (12.7) | 16,177 (18.4) |  | 7,566 (14.5) | 7,566 (14.5) |  |
|  | IMV | 1,792 (2.5) | 1,709 (1.9) |  | 834 (1.6) | 834 (1.6) |  |
| Omicron period | Early (Dec 2021-Dec 2022) | 43,243 (60.6) | 50,231 (57.0) | 0.07 | 31,656 (60.7) | 31,656 (60.7) | 0.00 |
|  | Later (Jan 2023-Dec 2024) | 28,131 (39.4) | 37,915 (43.0) |  | 20,484 (39.3) | 20,484 (39.3) |  |
| Data are presented as no. (%), unless otherwise indicated.  Abbreviations: COVID-19, coronavirus disease 2019; HFO/NIV, high-flow oxygen/non-invasive ventilation; ICF, intermediate care facility; ICU, intensive care unit; IMV, invasive mechanical ventilation; LFO, low-flow oxygen; NSOc, no supplemental oxygen charges; PS, propensity score; SMD, standardized mean difference; SNF, skilled nursing facility. | | | | | | | |

**eTable 8. Demographics of Patients Hospitalized for COVID-19 During December 2021-December 2024 Before and After IPTW (Elderly Population)**

| Characteristic | | Before IPTW | | | After IPTW | | |
| --- | --- | --- | --- | --- | --- | --- | --- |
|  |  | **No Remdesivir**  **n =71,374** | **Remdesivir**  **n = 88,146** | **Absolute SMD** | **No Remdesivir** | **Remdesivir** | **Absolute SMD** |
| Age group, years | 65–74 | 22,351 (31.3) | 29,041 (32.9) | 0.04 | 32.3 | 32.3 | 0.00 |
|  | 75–84 | 27,619 (38.7) | 33,904 (38.5) |  | 38.6 | 38.6 |  |
|  | ≥85 | 21,404 (30.0) | 25,201 (28.6) |  | 29.1 | 29.1 |  |
| Gender | Female | 37,495 (52.5) | 46,035 (52.2) | 0.01 | 52.2 | 52.2 | 0.00 |
| Race | White | 56,402 (79.0) | 70,755 (80.3) | 0.12 | 79.7 | 79.7 | 0.00 |
|  | Black | 9,251 (13.0) | 9,148 (10.4) |  | 11.4 | 11.5 |  |
|  | Asian | 1,367 (1.9) | 2,440 (2.8) |  | 2.4 | 2.4 |  |
|  | Other | 4,354 (6.1) | 5,803 (6.6) |  | 6.5 | 6.4 |  |
| Ethnicity | Hispanic | 4,661 (6.5) | 8,107 (9.2) | 0.07 | 7.9 | 8.0 | 0.00 |
|  | Non-Hispanic | 61,297 (85.9) | 74,392 (84.4) |  | 85.3 | 85.2 |  |
|  | Unknown | 5,416 (7.6) | 5,647 (6.4) |  | 6.8 | 6.8 |  |
| Primary payor | Commercial | 2,702 (3.8) | 3,980 (4.5) | 0.11 | 4.2 | 4.2 | 0.00 |
|  | Medicare | 65,471 (91.7) | 80,380 (91.2) |  | 91.4 | 91.4 |  |
|  | Medicaid | 928 (1.3) | 1,356 (1.5) |  | 1.4 | 1.4 |  |
|  | Other | 2,273 (3.2) | 2,430 (2.8) |  | 3.0 | 3.0 |  |
| Admission source | Transfer from SNF or ICF | 3,279 (4.6) | 4,509 (5.1) | 0.02 | 5.0 | 4.9 | 0.00 |
| Hospital size, number of beds | <100 | 5,616 (7.9) | 6,406 (7.3) | 0.12 | 7.7 | 7.6 | 0.03 |
|  | 100–199 | 12,233 (17.1) | 14,891 (16.9) |  | 16.9 | 16.9 |  |
|  | 200–299 | 15,077 (21.1) | 17,624 (20.0) |  | 20.3 | 20.6 |  |
|  | 300–399 | 14,158 (19.8) | 15,489 (17.6) |  | 18.2 | 18.3 |  |
|  | 400–499 | 7,601 (10.6) | 9,149 (10.4) |  | 10.8 | 10.7 |  |
|  | 500+ | 16,689 (23.4) | 24,587 (27.9) |  | 26.1 | 25.9 |  |
| Hospital location | Urban | 61,766 (86.5) | 78,173 (88.7) | 0.07 | 87.7 | 87.9 | 0.01 |
|  | Rural | 9,608 (13.5) | 9,973 (11.3) |  | 12.3 | 12.1 |  |
| Teaching hospital | | 30,931 (43.3) | 41,207 (46.7) | 0.07 | 45.3 | 45.4 | 0.00 |
| Region | Midwest | 17,657 (24.7) | 20,114 (22.8) | 0.19 | 23.9 | 24.0 | 0.00 |
|  | Northeast | 9,065 (12.7) | 16,920 (19.2) |  | 16.4 | 16.3 |  |
|  | South | 37,032 (51.9) | 40,130 (45.5) |  | 47.8 | 47.8 |  |
|  | West | 7,620 (10.7) | 10,982 (12.5) |  | 11.8 | 11.9 |  |
| Comorbid conditions | Obesity | 15,576 (21.8) | 19,880 (22.6) | 0.02 | 22.3 | 22.4 | 0.00 |
|  | Chronic pulmonary disease | 24,762 (34.7) | 35,565 (40.3) | 0.12 | 38.2 | 38.2 | 0.00 |
|  | Cardiovascular disease | 67,078 (94.0) | 82,167 (93.2) | 0.03 | 93.6 | 93.6 | 0.00 |
|  | Diabetes Mellitus | 29,032 (40.7) | 34,592 (39.2) | 0.03 | 40.0 | 40.0 | 0.00 |
|  | Renal disease | 27,204 (38.1) | 26,926 (30.5) | 0.16 | 34.2 | 34.3 | 0.00 |
|  | Cancer | 5,361 (7.5) | 7,687 (8.7) | 0.04 | 8.3 | 8.2 | 0.00 |
| Immunocompromising condition | | 11,642 (16.3) | 16,335 (18.5) | 0.06 | 17.8 | 17.8 | 0.00 |
| Hospital ward on admission | General ward | 61,495 (86.2) | 72,640 (82.4) | 0.10 | 84.1 | 84.0 | 0.00 |
|  | ICU/step down unit | 9,879 (13.8) | 15,506 (17.6) |  | 15.9 | 16.0 |  |
| Key diagnosis on admission | Sepsis | 346 (0.5) | 368 (0.4) | 0.01 | 0.5 | 0.5 | 0.00 |
|  | Pneumonia | 4,208 (5.9) | 5,492 (6.2) | 0.01 | 6.1 | 6.1 | 0.00 |
| Other COVID-19 treatments at baseline | Anticoagulants | 51,355 (72.0) | 68,179 (77.3) | 0.12 | 75.0 | 74.9 | 0.00 |
|  | Convalescent plasma | 19 (0.0) | 58 (0.1) | 0.51 | 0.0 | 0.0 | 0.00 |
|  | Corticosteroids | 43,066 (60.3) | 72,889 (82.7) | 0.02 | 73.0 | 73.2 | 0.00 |
|  | Baricitinib | 2,763 (3.9) | 3,043 (3.5) | 0.05 | 3.8 | 3.8 | 0.00 |
|  | Tocilizumab | 1,172 (1.6) | 2,057 (2.3) | 0.02 | 2.1 | 2.1 | 0.00 |
|  | Oral antivirals | 2,040 (2.9) | 314 (0.4) | 0.20 | 1.3 | 1.3 | 0.01 |
| Baseline supplemental oxygen requirements | NSOc | 40,795 (57.2) | 41,104 (46.6) | 0.21 | 51.0 | 50.8 | 0.00 |
|  | LFO | 19,736 (27.7) | 29,156 (33.1) |  | 30.6 | 30.8 |  |
|  | HFO/NIV | 9,051 (12.7) | 1,6177 (18.4) |  | 16.2 | 16.0 |  |
|  | IMV | 1,792 (2.5) | 1,709 (1.9) |  | 2.3 | 2.3 |  |
| Omicron period | Early (Dec 2021-Dec 2022) | 43,243 (60.6) | 50,231 (57.0) | 0.07 | 58.3 | 58.4 | 0.00 |
|  | Later (Jan 2023-Dec 2024) | 28,131 (39.4) | 37,915 (43.0) |  | 41.7 | 41.6 |  |
| Data is presented as n (%) before matching and as % after matching, unless otherwise indicated.  Abbreviations: COVID-19, coronavirus disease 2019; HFO/NIV, high-flow oxygen/non-invasive ventilation; ICF, intermediate care facility; ICU, intensive care unit; IMV, invasive mechanical ventilation; IPTW, inverse probability of treatment weighting; LFO, low-flow oxygen; NSOc, no supplemental oxygen charges; SMD, standardized mean difference; SNF, skilled nursing facility | | | | | | | |

**eTable 9. Unadjusted All-cause Inpatient Mortality Rates (PS matching without replacement, Elderly Population)**

|  | **Early Omicron (Dec 2021 - Dec 2022)** | | **Later Omicron (Jan 2023- Dec 2024)** | | **Overall (Dec 2021- Dec 2024)** | |
| --- | --- | --- | --- | --- | --- | --- |
|  | **No Remdesivir during the hospitalization** | **Remdesivir in the first two days** | **No Remdesivir during the hospitalization** | **Remdesivir in the first two days** | **No Remdesivir during the hospitalization** | **Remdesivir in the first two days** |
| **Overall Omicron** | **n=31656** | **n=31656** | **n=20484** | **n=20484** | **n=52140** | **n=52140** |
| 14-day mortality | 3919 (12.4) | 3036 (9.6) | 1447 (7.1) | 1092 (5.3) | 5366 (10.3) | 4128 (7.9) |
| 28-day mortality | 4943 (15.6) | 4036 (12.7) | 1672 (8.2) | 1281 (6.3) | 6615 (12.7) | 5317 (10.2) |
| **NSOc** | **n=15645** | **n=15645** | **n=11898** | **n=11898** | **n=27543** | **n=27543** |
| 14-day mortality | 1194 (7.6) | 993 (6.3) | 602 (5.1) | 471 (4.0) | 1796 (6.5) | 1464 (5.3) |
| 28-day mortality | 1474 (9.4) | 1273 (8.1) | 684 (5.7) | 540 (4.5) | 2158 (7.8) | 1813 (6.6) |
| **Any supplemental oxygen** | **n=16011** | **n=16011** | **n=8586** | **n=8586** | **n=24597** | **n=24597** |
| 14-day mortality | 2725 (17.0) | 2043 (12.8) | 845 (9.8) | 621 (7.2) | 3570 (14.5) | 2664 (10.8) |
| 28-day mortality | 3469 (21.7) | 2763 (17.3) | 988 (11.5) | 741 (8.6) | 4457 (18.1) | 3504 (14.2) |

Data is presented as n (%), unless otherwise indicated.

Abbreviations: PS, propensity score; NSOc, no supplemental oxygen charges.

**eTable 10. 14- and 28-day Mortality in Patients Hospitalized for COVID-19 Treated with Remdesivir Within the First 2 Days of Hospitalization vs Those Not Treated with Remdesivir During Hospitalization, by Maximal Supplemental Oxygen Requirements (IPTW, Elderly Population)**

|  | **aHR [95% CI]** | ***P* value** |
| --- | --- | --- |
| **14-day mortality** |  |  |
| Overall Omicron | 0.74 [0.71-0.77] | < 0.0001 |
| NSOc | 0.75 [0.70-0.80] | < 0.0001 |
| Any supplemental oxygen | 0.73 [0.70-0.77] | < 0.0001 |
| **28-day mortality** |  |  |
| Overall Omicron | 0.77 [0.74-0.80] | < 0.0001 |
| NSOc | 0.78 [0.73-0.83] | < 0.0001 |
| Any supplemental oxygen | 0.76 [0.72-0.79] | < 0.0001 |

Cox proportional hazards model was used to derive estimates adjusted admission month, and time-varying treatment with other COVID-19 medications (baricitinib, tocilizumab, oral antivirals).

Abbreviations: aHR, adjusted hazard ratio; CI, confidence interval; COVID-19, coronavirus disease 2019; IPTW, inverse probability of treatment weighting; NSOc, no supplemental oxygen charges.

**eTable 11. 14- and 28-day Mortality in Patients Hospitalized for COVID-19 Treated with Remdesivir Within the First 2 Days of Hospitalization vs Those Not Treated with Remdesivir Within the First 2 Days, by Maximal Supplemental Oxygen Requirements (PS matching, Elderly Population)**

|  | **N** | **aHR [95% CI]** | ***P* value** |
| --- | --- | --- | --- |
| **14-day mortality** |  |  |  |
| Overall Omicron | 111,726 | 0.74 [0.71-0.77] | < 0.0001 |
| NSOc | 58,424 | 0.77 [0.71-0.82] | < 0.0001 |
| Any supplemental oxygen | 53,302 | 0.73 [0.69-0.77] | < 0.0001 |
| **28-day mortality** |  |  |  |
| Overall Omicron | 111,726 | 0.76 [0.73-0.79] | < 0.0001 |
| NSOc | 58,424 | 0.78 [0.73-0.83] | < 0.0001 |
| Any supplemental oxygen | 53,302 | 0.75 [0.71-0.79] | < 0.0001 |

Cox proportional hazards model was used to derive estimates adjusted admission month, and time-varying treatment with other COVID-19 medications (baricitinib, tocilizumab, oral antivirals).

Abbreviations: aHR, adjusted hazard ratio; CI, confidence interval; COVID-19, coronavirus disease 2019; NSOc, no supplemental oxygen charges; PS, propensity score.

**eTable 12. Demographics of Patients Hospitalized for COVID-19 from December 2021-December 2024 Before and After PS matching (Pneumonia population)**

| Characteristic | | Before PS matching | | | After PS matching | | |
| --- | --- | --- | --- | --- | --- | --- | --- |
|  |  | **No Remdesivir**  **n = 54,735** | **Remdesivir**  **n = 86,496** | **Absolute SMD** | **No Remdesivir**  **n = 44,754** | **Remdesivir**  **n = 44,754** | **Absolute SMD** |
| Age group, years | 18–49 | 4,528 (8.3) | 8,484 (9.8) | 0.08 | 3,471 (7.8) | 3,471 (7.8) | 0.00 |
|  | 50–64 | 11,473 (21.0) | 19,056 (22.0) |  | 9,320 (20.8) | 9,320 (20.8) |  |
|  | ≥65 | 38,734 (70.8) | 58,956 (68.2) |  | 31,963 (71.4) | 31,963 (71.4) |  |
| Gender | Female | 27,144 (49.6) | 43,328 (50.1) | 0.01 | 22,221 (49.7) | 22,199 (49.6) | 0.00 |
| Race | White | 41,935 (76.6) | 66,607 (77.0) | 0.09 | 34,766 (77.7) | 34,842 (77.9) | 0.00 |
|  | Black | 8,026 (14.7) | 10,863 (12.6) |  | 6,002 (13.4) | 5,919 (13.2) |  |
|  | Asian | 919 (1.7) | 2,028 (2.3) |  | 806 (1.8) | 796 (1.8) |  |
|  | Other | 3,855 (7.0) | 6,998 (8.1) |  | 3,180 (7.1) | 3,197 (7.1) |  |
| Ethnicity | Hispanic | 4,368 (8.0) | 9,341 (10.8) | 0.11 | 3,592 (8.0) | 3,605 (8.1) | 0.00 |
|  | Non-Hispanic | 46,308 (84.6) | 71,462 (82.6) |  | 38,024 (85.0) | 37,966 (84.8) |  |
|  | Unknown | 4,059 (7.4) | 5,693 (6.6) |  | 3,138 (7.0) | 3,183 (7.1) |  |
| Primary payor | Commercial | 7,647 (14.0) | 14,213 (16.4) | 0.07 | 6,591 (14.7) | 6,495 (14.5) | 0.00 |
|  | Medicare | 39,622 (72.4) | 60,020 (69.4) |  | 32,327 (72.2) | 32,303 (72.2) |  |
|  | Medicaid | 4,425 (8.1) | 7,766 (9.0) |  | 3,456 (7.7) | 3,510 (7.8) |  |
|  | Other | 3,041 (5.6) | 4,497 (5.2) |  | 2,380 (5.3) | 2,446 (5.5) |  |
| Admission source | Transfer from SNF or ICF | 1,825 (3.3) | 3,120 (3.6) | 0.01 | 1,539 (3.4) | 1,545 (3.5) | 0.00 |
| Hospital size, number of beds | <100 | 4,178 (7.6) | 6,451 (7.5) | 0.09 | 3,515 (7.9) | 3,422 (7.6) | 0.06 |
|  | 100–199 | 9,490 (17.3) | 15,490 (17.9) |  | 7,818 (17.5) | 7,911 (17.7) |  |
|  | 200–299 | 11,551 (21.1) | 17,590 (20.3) |  | 9,538 (21.3) | 9,370 (20.9) |  |
|  | 300–399 | 10,588 (19.3) | 15,357 (17.8) |  | 8,660 (19.4) | 8,467 (18.9) |  |
|  | 400–499 | 6,117 (11.2) | 8,848 (10.2) |  | 4,789 (10.7) | 5,150 (11.5) |  |
|  | 500+ | 12,811 (23.4) | 22,760 (26.3) |  | 10,434 (23.3) | 10,434 (23.3) |  |
| Hospital location | Urban | 47,230 (86.3) | 76,082 (88.0) | 0.05 | 38,706 (86.5) | 38,726 (86.5) | 0.00 |
|  | Rural | 7,505 (13.7) | 10,414 (12.0) |  | 6,048 (13.5) | 6,028 (13.5) |  |
| Teaching hospital | | 23,776 (43.4) | 38,987 (45.1) | 0.03 | 19,247 (43.0) | 19,447 (43.5) | 0.01 |
| Region | Midwest | 14,136 (25.8) | 20,770 (24.0) | 0.22 | 11,688 (26.1) | 11,485 (25.7) | 0.00 |
|  | Northeast | 5,176 (9.5) | 13,848 (16.0) |  | 4,632 (10.3) | 4,647 (10.4) |  |
|  | South | 28,831 (52.7) | 40,558 (46.9) |  | 22,830 (51.0) | 23,006 (51.4) |  |
|  | West | 6,592 (12.0) | 11,320 (13.1) |  | 5,604 (12.5) | 5,616 (12.5) |  |
| Comorbid conditions | Obesity | 16,662 (30.4) | 27,432 (31.7) | 0.03 | 13,889 (31.0) | 13,843 (30.9) | 0.00 |
|  | Chronic pulmonary disease | 20,066 (36.7) | 34,251 (39.6) | 0.06 | 16,955 (37.9) | 17,058 (38.1) | 0.00 |
|  | Cardiovascular disease | 48,659 (88.9) | 74,743 (86.4) | 0.08 | 39,469 (88.2) | 39,519 (88.3) | 0.00 |
|  | Diabetes Mellitus | 22,805 (41.7) | 34,130 (39.5) | 0.04 | 18,281 (40.8) | 18,215 (40.7) | 0.00 |
|  | Renal disease | 19,526 (35.7) | 22,008 (25.4) | 0.22 | 14,651 (32.7) | 14,450 (32.3) | 0.01 |
|  | Cancer | 4,013 (7.3) | 6,534 (7.6) | 0.01 | 3,307 (7.4) | 3,369 (7.5) | 0.01 |
| Immunocompromising condition | | 9,561 (17.5) | 15,368 (17.8) | 0.01 | 7,827 (17.5) | 7,881 (17.6) | 0.00 |
| Hospital ward on admission | General ward | 44,735 (81.7) | 69,839 (80.7) | 0.03 | 36,823 (82.3) | 36,940 (82.5) | 0.01 |
|  | ICU/step down unit | 10,000 (18.3) | 16,657 (19.3) |  | 7,931 (17.7) | 7,814 (17.5) |  |
| Key Diagnosis on admission | Sepsis | 279 (0.5) | 362 (0.4) | 0.01 | 213 (0.5) | 200 (0.4) | 0.00 |
|  | Pneumonia | 4,663 (8.5) | 6,380 (7.4) | 0.04 | 3,614 (8.1) | 3,620 (8.1) | 0.00 |
| Other COVID-19 treatments at baseline | Anticoagulants | 41,046 (75.0) | 69,891 (80.8) | 0.14 | 34,802 (77.8) | 34,932 (78.1) | 0.01 |
|  | Convalescent plasma | 31 (0.1) | 100 (0.1) | 0.39 | 29 (0.1) | 24 (0.1) | 0.00 |
|  | Corticosteroids | 42,642 (77.9) | 79,205 (91.6) | 0.02 | 38,919 (87.0) | 38,901 (86.9) | 0.00 |
|  | Baricitinib | 4,306 (7.9) | 5,627 (6.5) | 0.04 | 3,585 (8.0) | 3,613 (8.1) | 0.00 |
|  | Tocilizumab | 1,998 (3.7) | 3,849 (4.4) | 0.05 | 1,793 (4.0) | 1,816 (4.1) | 0.00 |
|  | Oral antivirals | 719 (1.3) | 178 (0.2) | 0.13 | 101 (0.2) | 100 (0.2) | 0.00 |
| Baseline supplemental oxygen requirements | NSOc | 23,373 (42.7) | 32,398 (37.5) | 0.15 | 18,190 (40.6) | 18,190 (40.6) | 0.00 |
|  | LFO | 18,447 (33.7) | 31,329 (36.2) |  | 15,996 (35.7) | 15,996 (35.7) |  |
|  | HFO/NIV | 10,341 (18.9) | 20,075 (23.2) |  | 9,075 (20.3) | 9,075 (20.3) |  |
|  | IMV | 2,574 (4.7) | 2,694 (3.1) |  | 1,493 (3.3) | 1,493 (3.3) |  |
| Omicron period | Early (Dec 2021-Dec 2022) | 40,226 (73.5) | 61,131 (70.7) | 0.06 | 33,563 (75.0) | 33,563 (75.0) | 0.00 |
|  | Later (Jan 2023-Dec 2024) | 14,509 (26.5) | 25,365 (29.3) |  | 11,191 (25.0) | 11,191 (25.0) |  |
| Data is presented as no. (%), unless otherwise indicated.  Abbreviations: COVID-19, coronavirus disease 2019; HFO/NIV, high flow oxygen/non-invasive ventilation; ICF, intermediate care facility; ICU, intensive care unit; IMV, invasive mechanical ventilation; LFO, low flow oxygen; NSOc, no supplemental oxygen charges; PS, propensity score; SMD, standardized mean difference; SNF, skilled nursing facility | | | | | | | |

**eTable 13. Demographics of Patients Hospitalized for COVID-19 During December 2021-December 2024 Before and After IPTW (Pneumonia Population)**

| Characteristic | | Before IPTW | | | After IPTW | | |
| --- | --- | --- | --- | --- | --- | --- | --- |
|  |  | **No Remdesivir**  **n = 54,735** | **Remdesivir**  **n = 86,496** | **Absolute SMD** | **No Remdesivir** | **Remdesivir** | **Absolute SMD** |
| Age group, years | 18–49 | 4,528 (8.3) | 8,484 (9.8) | 0.08 | 9.3 | 9.2 | 0.00 |
|  | 50–64 | 11,473 (21.0) | 19,056 (22.0) |  | 21.7 | 21.6 |  |
|  | ≥65 | 38,734 (70.8) | 58,956 (68.2) |  | 69.0 | 69.2 |  |
| Gender | Female | 27,144 (49.6) | 43,328 (50.1) | 0.01 | 49.8 | 49.8 | 0.00 |
| Race | White | 41,935 (76.6) | 66,607 (77.0) | 0.09 | 77.0 | 77.0 | 0.00 |
|  | Black | 8,026 (14.7) | 10,863 (12.6) |  | 13.2 | 13.3 |  |
|  | Asian | 919 (1.7) | 2,028 (2.3) |  | 2.1 | 2.1 |  |
|  | Other | 3,855 (7.0) | 6,998 (8.1) |  | 7.8 | 7.7 |  |
| Ethnicity | Hispanic | 4,368 (8.0) | 9,341 (10.8) | 0.11 | 9.7 | 9.7 | 0.00 |
|  | Non-Hispanic | 46,308 (84.6) | 71,462 (82.6) |  | 83.5 | 83.5 |  |
|  | Unknown | 4,059 (7.4) | 5,693 (6.6) |  | 6.8 | 6.8 |  |
| Primary payor | Commercial | 7,647 (14.0) | 14,213 (16.4) | 0.07 | 15.5 | 15.5 | 0.05 |
|  | Medicare | 39,622 (72.4) | 60,020 (69.4) |  | 70.4 | 70.5 |  |
|  | Medicaid | 4,425 (8.1) | 7,766 (9.0) |  | 8.7 | 8.7 |  |
|  | Other | 3,041 (5.6) | 4,497 (5.2) |  | 5.3 | 5.3 |  |
| Admission source | Transfer from SNF or ICF | 1,825 (3.3) | 3,120 (3.6) | 0.01 | 3.5 | 3.5 | 0.00 |
| Hospital size, number of beds | <100 | 4,178 (7.6) | 6,451 (7.5) | 0.09 | 7.8 | 7.6 | 0.03 |
|  | 100–199 | 9,490 (17.3) | 15,490 (17.9) |  | 17.7 | 17.7 |  |
|  | 200–299 | 11,551 (21.1) | 17,590 (20.3) |  | 20.5 | 20.7 |  |
|  | 300–399 | 10,588 (19.3) | 15,357 (17.8) |  | 18.1 | 18.3 |  |
|  | 400–499 | 6,117 (11.2) | 8,848 (10.2) |  | 10.8 | 10.7 |  |
|  | 500+ | 12,811 (23.4) | 2,2760 (26.3) |  | 25.2 | 25.1 |  |
| Hospital location | Urban | 47,230 (86.3) | 7,6082 (88.0) | 0.05 | 87.2 | 87.4 | 0.01 |
|  | Rural | 7,505 (13.7) | 10,414 (12.0) |  | 12.8 | 12.6 |  |
| Teaching hospital | | 23,776 (43.4) | 38,987 (45.1) | 0.03 | 44.5 | 44.5 | 0.00 |
| Region | Midwest | 14,136 (25.8) | 20,770 (24.0) | 0.22 | 24.9 | 24.9 | 0.04 |
|  | Northeast | 5,176 (9.5) | 13,848 (16.0) |  | 13.6 | 13.5 |  |
|  | South | 28,831 (52.7) | 40,558 (46.9) |  | 48.6 | 48.7 |  |
|  | West | 6,592 (12.0) | 11,320 (13.1) |  | 12.9 | 12.9 |  |
| Comorbid conditions | Obesity | 16,662 (30.4) | 27,432 (31.7) | 0.03 | 31.3 | 31.3 | 0.00 |
|  | Chronic pulmonary disease | 20,066 (36.7) | 34,251 (39.6) | 0.06 | 38.7 | 38.6 | 0.00 |
|  | Cardiovascular disease | 48,659 (88.9) | 74,743 (86.4) | 0.08 | 87.4 | 87.4 | 0.00 |
|  | Diabetes Mellitus | 22,805 (41.7) | 34,130 (39.5) | 0.04 | 40.2 | 40.3 | 0.00 |
|  | Renal disease | 19,526 (35.7) | 22,008 (25.4) | 0.22 | 29.5 | 29.5 | 0.00 |
|  | Cancer | 4,013 (7.3) | 6,534 (7.6) | 0.01 | 7.5 | 7.5 | 0.00 |
| Immunocompromising condition | | 9,561 (17.5) | 15,368 (17.8) | 0.01 | 17.8 | 17.7 | 0.00 |
| Hospital ward on admission | General ward | 44,735 (81.7) | 69,839 (80.7) | 0.03 | 81.2 | 81.1 | 0.00 |
|  | ICU/step down unit | 10,000 (18.3) | 16,657 (19.3) |  | 18.8 | 18.9 |  |
| Key diagnosis on admission | Sepsis | 279 (0.5) | 362 (0.4) | 0.01 | 0.5 | 0.5 | 0.00 |
|  | Pneumonia | 4,663 (8.5) | 6,380 (7.4) | 0.04 | 7.7 | 7.8 | 0.00 |
| Other COVID-19 treatments at baseline | Anticoagulants | 41,046 (75.0) | 69,891 (80.8) | 0.14 | 78.7 | 78.5 | 0.00 |
|  | Convalescent plasma | 31 (0.1) | 100 (0.1) | 0.39 | 0.1 | 0.1 | 0.00 |
|  | Corticosteroids | 42,642 (77.9) | 79,205 (91.6) | 0.02 | 86.3 | 86.3 | 0.00 |
|  | Baricitinib | 4,306 (7.9) | 5,627 (6.5) | 0.04 | 7.2 | 7.2 | 0.00 |
|  | Tocilizumab | 1,998 (3.7) | 3,849 (4.4) | 0.05 | 4.3 | 4.2 | 0.00 |
|  | Oral antivirals | 719 (1.3) | 178 (0.2) | 0.13 | 0.6 | 0.7 | 0.00 |
| Baseline supplemental oxygen requirements | NSOc | 23,373 (42.7) | 32,398 (37.5) | 0.15 | 39.3 | 39.3 | 0.00 |
|  | LFO | 18,447 (33.7) | 31,329 (36.2) |  | 35.1 | 35.2 |  |
|  | HFO/NIV | 10,341 (18.9) | 20,075 (23.2) |  | 21.8 | 21.6 |  |
|  | IMV | 2,574 (4.7) | 2,694 (3.1) |  | 3.8 | 3.8 |  |
| Omicron period | Early (Dec 2021-Dec 2022) | 40,226 (73.5) | 61,131 (70.7) | 0.06 | 71.7 | 71.7 | 0.00 |
|  | Later (Jan 2023-Dec 2024) | 14,509 (26.5) | 25,365 (29.3) |  | 28.3 | 28.3 |  |
| Data is presented as n (%) before matching and as % after matching, unless otherwise indicated. Abbreviations: COVID-19, coronavirus disease 2019; HFO/NIV, high-flow oxygen/non-invasive ventilation; ICF, intermediate care facility; ICU, intensive care unit; IMV, invasive mechanical ventilation; IPTW, inverse probability of treatment weighting; LFO, low-flow oxygen; NSOc, no supplemental oxygen charges; SMD, standardized mean difference; SNF, skilled nursing facility | | | | | | | |

**eTable 14. Unadjusted All-cause Inpatient Mortality Rates (PS matching without replacement, Pneumonia Population)**

|  | **Early Omicron (Dec 2021 - Dec 2022)** | | **Later Omicron (Jan 2023- Dec 2024)** | | **Overall (Dec 2021- Dec 2024)** | |
| --- | --- | --- | --- | --- | --- | --- |
|  | **No Remdesivir during the hospitalization** | **Remdesivir in the first two days** | **No Remdesivir during the hospitalization** | **Remdesivir in the first two days** | **No Remdesivir during the hospitalization** | **Remdesivir in the first two days** |
| **Overall Omicron** | **n=33563** | **n=33563** | **n=11191** | **n=11191** | **n=44754** | **n=44754** |
| 14-day mortality | 4116 (12.3) | 3226 (9.6) | 1028 (9.2) | 825 (7.4) | 5144 (11.5) | 4051 (9.1) |
| 28-day mortality | 5460 (16.3) | 4503 (13.4) | 1231 (11.0) | 992 (8.9) | 6691 (15.0) | 5495 (12.3) |
| **NSOc** | **n=13191** | **n=13191** | **n=4999** | **n=4999** | **n=18190** | **n=18190** |
| 14-day mortality | 1051 (8.0) | 832 (6.3) | 308 (6.2) | 262 (5.2) | 1359 (7.5) | 1094 (6.0) |
| 28-day mortality | 1358 (10.3) | 1152 (8.7) | 362 (7.2) | 309 (6.2) | 1720 (9.5) | 1461 (8.0) |
| **Any supplemental oxygen** | **n=20372** | **n=20372** | **n=6192** | **n=6192** | **n=26564** | **n=26564** |
| 14-day mortality | 3065 (15.0) | 2394 (11.8) | 720 (11.6) | 563 (9.1) | 3785 (14.2) | 2957 (11.1) |
| 28-day mortality | 4102 (20.1) | 3351 (16.4) | 869 (14.0) | 683 (11.0) | 4971 (18.7) | 4034 (15.2) |

Data is presented as n (%), unless otherwise indicated.

Abbreviations: PS, propensity score; NSOc, no supplemental oxygen charges.

**eTable 15. 14- and 28-day Mortality in Patients Hospitalized for COVID-19 Treated with Remdesivir Within the First 2 Days of Hospitalization vs Those Not Treated with Remdesivir During Hospitalization, by Maximal Supplemental Oxygen Requirements (IPTW, Pneumonia Population)**

|  | **aHR [95% CI]** | ***P* value** |
| --- | --- | --- |
| **14-day mortality** |  |  |
| Overall Omicron | 0.75 [0.72-0.78] | < 0.0001 |
| NSOc | 0.76 [0.71-0.81] | < 0.0001 |
| Any supplemental oxygen | 0.75 [0.71-0.78] | < 0.0001 |
| **28-day mortality** |  |  |
| Overall Omicron | 0.78 [0.75-0.81] | < 0.0001 |
| NSOc | 0.78 [0.73-0.83] | < 0.0001 |
| Any supplemental oxygen | 0.77 [0.74-0.81] | < 0.0001 |

Cox proportional hazards model was used to derive estimates adjusted for admission month and time-varying treatment with other COVID-19 medications (baricitinib, tocilizumab, oral antivirals).

Abbreviations: aHR, adjusted hazard ratio; CI, confidence interval; COVID-19, coronavirus disease 2019; IPTW, inverse probability of treatment weighting; NSOc, no supplemental oxygen charges.

**eTable 16. 14- and 28-day Mortality in Patients Hospitalized for COVID-19 Treated with Remdesivir Within the First 2 Days of Hospitalization vs Those Not Treated with Remdesivir Within the First 2 Days, by Maximal Supplemental Oxygen Requirements (PS matching, Pneumonia Population)**

|  | **N** | **aHR [95% CI]** | ***P* value** |
| --- | --- | --- | --- |
| **14-day mortality** |  |  |  |
| Overall Omicron | 98,164 | 0.75 [0.72-0.78] | < 0.0001 |
| NSOc | 39,966 | 0.72 [0.66-0.77] | < 0.0001 |
| Any supplemental oxygen | 58,198 | 0.76 [0.72-0.80] | < 0.0001 |
| **28-day mortality** |  |  |  |
| Overall Omicron | 98,164 | 0.77 [0.74-0.80] | < 0.0001 |
| NSOc | 39,966 | 0.74 [0.69-0.79] | < 0.0001 |
| Any supplemental oxygen | 58,198 | 0.77 [0.74-0.81] | < 0.0001 |

Cox proportional hazards model was used to derive estimates adjusted for admission month and time-varying treatment with other COVID-19 medications (baricitinib, tocilizumab, oral antivirals).

Abbreviations: aHR, adjusted hazard ratio; CI, confidence interval; COVID-19, coronavirus disease 2019; NSOc, no supplemental oxygen charges; PS, propensity score.

**eTable 17. Demographics of Patients Hospitalized for COVID-19 from December 2021-December 2024 Before and After PS matching (COPD population)**

| Characteristic | | Before PS matching | | | After PS matching | | |
| --- | --- | --- | --- | --- | --- | --- | --- |
|  |  | **No Remdesivir**  **n = 26,003** | **Remdesivir**  **n = 38,457** | **Absolute SMD** | **No Remdesivir**  **n = 20,408** | **Remdesivir**  **n = 20,408** | **Absolute SMD** |
| Age group, years | 18–49 | 517 (2.0) | 821 (2.1) | 0.03 | 215 (1.1) | 215 (1.1) | 0.00 |
|  | 50–64 | 4,863 (18.7) | 7,676 (20.0) |  | 3,530 (17.3) | 3,530 (17.3) |  |
|  | ≥65 | 20,623 (79.3) | 29,960 (77.9) |  | 16,663 (81.6) | 16,663 (81.6) |  |
| Gender | Female | 14,068 (54.1) | 20,809 (54.1) | 0.00 | 11,048 (54.1) | 11,099 (54.4) | 0.01 |
| Race | White | 20,933 (80.5) | 31,174 (81.1) | 0.11 | 16,632 (81.5) | 16,712 (81.9) | 0.03 |
|  | Black | 3,620 (13.9) | 4,688 (12.2) |  | 2,609 (12.8) | 2,547 (12.5) |  |
|  | Asian | 202 (0.8) | 503 (1.3) |  | 175 (0.9) | 164 (0.8) |  |
|  | Other | 1,248 (4.8) | 2,092 (5.4) |  | 992 (4.9) | 985 (4.8) |  |
| Ethnicity | Hispanic | 1,165 (4.5) | 2,365 (6.1) | 0.10 | 935 (4.6) | 921 (4.5) | 0.04 |
|  | Non-Hispanic | 22,872 (88.0) | 33,555 (87.3) |  | 18,062 (88.5) | 18,047 (88.4) |  |
|  | Unknown | 1,966 (7.6) | 2,537 (6.6) |  | 1,411 (6.9) | 1,440 (7.1) |  |
| Primary payor | Commercial | 1,641 (6.3) | 3,092 (8.0) | 0.08 | 1,311 (6.4) | 1,322 (6.5) | 0.05 |
|  | Medicare | 21,229 (81.6) | 30,713 (79.9) |  | 16,886 (82.7) | 1,6806 (82.4) |  |
|  | Medicaid | 1,962 (7.5) | 3,080 (8.0) |  | 1,369 (6.7) | 1,375 (6.7) |  |
|  | Other | 1,171 (4.5) | 1,572 (4.1) |  | 842 (4.1) | 905 (4.4) |  |
| Admission source | Transfer from SNF or ICF | 1,071 (4.1) | 1,687 (4.4) | 0.01 | 862 (4.2) | 860 (4.2) | 0.00 |
| Hospital size, number of beds | <100 | 1,956 (7.5) | 3,052 (7.9) | 0.08 | 1,614 (7.9) | 1,575 (7.7) | 0.04 |
|  | 100–199 | 4,550 (17.5) | 6,518 (16.9) |  | 3,468 (17.0) | 3,507 (17.2) |  |
|  | 200–299 | 5,528 (21.3) | 7,863 (20.4) |  | 4,439 (21.8) | 4,337 (21.3) |  |
|  | 300–399 | 5,256 (20.2) | 7,033 (18.3) |  | 4,103 (20.1) | 4,113 (20.2) |  |
|  | 400–499 | 2,674 (10.3) | 3,800 (9.9) |  | 2,090 (10.2) | 2,182 (10.7) |  |
|  | 500+ | 6,039 (23.2) | 10,191 (26.5) |  | 4,694 (23.0) | 4,694 (23.0) |  |
| Hospital location | Urban | 21,997 (84.6) | 33,274 (86.5) | 0.05 | 17,345 (85.0) | 17,307 (84.8) | 0.01 |
|  | Rural | 4,006 (15.4) | 5,183 (13.5) |  | 3,063 (15.0) | 3,101 (15.2) |  |
| Teaching hospital | | 11,204 (43.1) | 17,327 (45.1) | 0.04 | 8,712 (42.7) | 8,764 (42.9) | 0.01 |
| Region | Midwest | 7,049 (27.1) | 9,599 (25.0) | 0.22 | 5,530 (27.1) | 5,391 (26.4) | 0.05 |
|  | Northeast | 2,701 (10.4) | 6,640 (17.3) |  | 2,300 (11.3) | 2,385 (11.7) |  |
|  | South | 13,785 (53.0) | 17,983 (46.8) |  | 10,502 (51.5) | 10,577 (51.8) |  |
|  | West | 2,468 (9.5) | 4,235 (11.0) |  | 2,076 (10.2) | 2,055 (10.1) |  |
| Comorbid conditions | Obesity | 7,527 (28.9) | 11,287 (29.3) | 0.01 | 5,834 (28.6) | 5,808 (28.5) | 0.00 |
|  | Cardiovascular disease | 24,451 (94.0) | 35,602 (92.6) | 0.06 | 19,147 (93.8) | 19,141 (93.8) | 0.00 |
|  | Diabetes Mellitus | 10,736 (41.3) | 14,991 (39.0) | 0.05 | 8,240 (40.4) | 8,164 (40.0) | 0.01 |
|  | Renal disease | 9,579 (36.8) | 10,740 (27.9) | 0.19 | 6,983 (34.2) | 6,899 (33.8) | 0.01 |
|  | Cancer | 2,030 (7.8) | 3,242 (8.4) | 0.02 | 1,621 (7.9) | 1660 (8.1) | 0.01 |
| Immunocompromising condition | | 4,786 (18.4) | 7,620 (19.8) | 0.04 | 3,861 (18.9) | 3,867 (18.9) | 0.00 |
| Hospital ward on admission | General ward | 21,569 (82.9) | 31,220 (81.2) | 0.05 | 16,914 (82.9) | 16,957 (83.1) | 0.01 |
|  | ICU/step down unit | 4,434 (17.1) | 7,237 (18.8) |  | 3,494 (17.1) | 3,451 (16.9) |  |
| Key Diagnosis on admission | Sepsis | 138 (0.5) | 139 (0.4) | 0.03 | 90 (0.4) | 88 (0.4) | 0.00 |
|  | Pneumonia | 2,023 (7.8) | 2,803 (7.3) | 0.02 | 1,532 (7.5) | 1531 (7.5) | 0.00 |
| Other COVID-19 treatments at baseline | Anticoagulants | 19,089 (73.4) | 30,148 (78.4) | 0.12 | 15,495 (75.9) | 15,561 (76.2) | 0.01 |
|  | Convalescent plasma | 1 (0.0) | 21 (0.1) | 0.38 | 1 (0.0) | 0 (0.0) | 0.00 |
|  | Corticosteroids | 20,400 (78.5) | 35,264 (91.7) | 0.03 | 17,939 (87.9) | 17,931 (87.9) | 0.01 |
|  | Baricitinib | 1,356 (5.2) | 1,505 (3.9) | 0.04 | 978 (4.8) | 986 (4.8) | 0.00 |
|  | Tocilizumab | 476 (1.8) | 915 (2.4) | 0.06 | 417 (2.0) | 415 (2.0) | 0.00 |
|  | Oral antivirals | 590 (2.3) | 106 (0.3) | 0.18 | 48 (0.2) | 45 (0.2) | 0.00 |
| Baseline supplemental oxygen requirements | NSOc | 10,269 (39.5) | 12,735 (33.1) | 0.18 | 7,584 (37.2) | 7,584 (37.2) | 0.00 |
|  | LFO | 9,191 (35.3) | 14,094 (36.6) |  | 7,518 (36.8) | 7,518 (36.8) |  |
|  | HFO/NIV | 5,452 (21.0) | 10,479 (27.2) |  | 4,645 (22.8) | 4,645 (22.8) |  |
|  | IMV | 1,091 (4.2) | 1,149 (3.0) |  | 661 (3.2) | 661 (3.2) |  |
| Omicron period | Early (Dec 2021-Dec 2022) | 15,794 (60.7) | 22,281 (57.9) | 0.06 | 12,678 (62.1) | 12,678 (62.1) | 0.00 |
|  | Later (Jan 2023-Dec 2024) | 10,209 (39.3) | 16,176 (42.1) |  | 7,730 (37.9) | 7,730 (37.9) |  |
| Data are presented as no. (%), unless otherwise indicated.  Abbreviations: COPD, chronic obstructive pulmonary disease; COVID-19, coronavirus disease 2019; HFO/NIV, high-flow oxygen/non-invasive ventilation; ICF, intermediate care facility; ICU, intensive care unit; IMV, invasive mechanical ventilation; LFO, low-flow oxygen; NSOc, no supplemental oxygen charges; PS, propensity score; SMD, standardized mean difference; SNF, skilled nursing facility | | | | | | | |

**eTable 18. Demographics of Patients Hospitalized for COVID-19 During December 2021-December 2024 Before and After IPTW (COPD population)**

| Characteristic | | Before IPTW | | | After IPTW | | |
| --- | --- | --- | --- | --- | --- | --- | --- |
|  |  | **No Remdesivir**  **n = 26,003** | **Remdesivir**  **n = 38,457** | **Absolute SMD** | **No Remdesivir** | **Remdesivir** | **Absolute SMD** |
| Age group, years | 18–49 | 517 (2.0) | 821 (2.1) | 0.03 | 2.1 | 2.1 | 0.03 |
|  | 50–64 | 4,863 (18.7) | 7,676 (20.0) |  | 19.5 | 19.4 |  |
|  | ≥65 | 20,623 (79.3) | 29,960 (77.9) |  | 78.4 | 78.5 |  |
| Gender | Female | 14,068 (54.1) | 20,809 (54.1) | 0.00 | 54.1 | 54.0 | 0.00 |
| Race | White | 20,933 (80.5) | 31,174 (81.1) | 0.11 | 80.8 | 80.9 | 0.00 |
|  | Black | 3,620 (13.9) | 4,688 (12.2) |  | 12.7 | 12.8 |  |
|  | Asian | 202 (0.8) | 503 (1.3) |  | 1.1 | 1.1 |  |
|  | Other | 1,248 (4.8) | 2,092 (5.4) |  | 5.4 | 5.2 |  |
| Ethnicity | Hispanic | 1,165 (4.5) | 2,365 (6.1) | 0.10 | 5.6 | 5.5 | 0.06 |
|  | Non-Hispanic | 22,872 (88.0) | 33,555 (87.3) |  | 87.6 | 87.6 |  |
|  | Unknown | 1,966 (7.6) | 2,537 (6.6) |  | 6.8 | 6.9 |  |
| Primary payor | Commercial | 1,641 (6.3) | 3,092 (8.0) | 0.08 | 7.3 | 7.3 | 0.00 |
|  | Medicare | 21,229 (81.6) | 30,713 (79.9) |  | 80.5 | 80.6 |  |
|  | Medicaid | 1,962 (7.5) | 3,080 (8.0) |  | 7.9 | 7.8 |  |
|  | Other | 1,171 (4.5) | 1,572 (4.1) |  | 4.3 | 4.3 |  |
| Admission source | Transfer from SNF or ICF | 1,071 (4.1) | 1,687 (4.4) | 0.01 | 4.4 | 4.3 | 0.00 |
| Hospital size, number of beds | <100 | 1,956 (7.5) | 3,052 (7.9) | 0.08 | 8.0 | 7.8 | 0.00 |
|  | 100–199 | 4,550 (17.5) | 6,518 (16.9) |  | 17.1 | 17.1 |  |
|  | 200–299 | 5,528 (21.3) | 7,863 (20.4) |  | 20.6 | 20.8 |  |
|  | 300–399 | 5,256 (20.2) | 7,033 (18.3) |  | 18.9 | 18.9 |  |
|  | 400–499 | 2,674 (10.3) | 3,800 (9.9) |  | 10.2 | 10.2 |  |
|  | 500+ | 6,039 (23.2) | 10,191 (26.5) |  | 25.3 | 25.1 |  |
| Hospital location | Urban | 21,997 (84.6) | 33,274 (86.5) | 0.05 | 85.7 | 85.8 | 0.00 |
|  | Rural | 4,006 (15.4) | 5,183 (13.5) |  | 14.3 | 14.2 |  |
| Teaching hospital | | 11,204 (43.1) | 17,327 (45.1) | 0.04 | 44.4 | 44.4 | 0.00 |
| Region | Midwest | 7,049 (27.1) | 9,599 (25.0) | 0.22 | 25.9 | 26.0 | 0.00 |
|  | Northeast | 2,701 (10.4) | 6,640 (17.3) |  | 14.7 | 14.6 |  |
|  | South | 13,785 (53.0) | 17,983 (46.8) |  | 48.9 | 48.9 |  |
|  | West | 2,468 (9.5) | 4,235 (11.0) |  | 10.6 | 10.6 |  |
| Comorbid conditions | Obesity | 7,527 (28.9) | 11,287 (29.3) | 0.01 | 29.2 | 29.2 | 0.00 |
|  | Cardiovascular disease | 24,451 (94.0) | 35,602 (92.6) | 0.06 | 93.1 | 93.2 | 0.00 |
|  | Diabetes Mellitus | 10,736 (41.3) | 14,991 (39.0) | 0.05 | 39.9 | 39.9 | 0.00 |
|  | Renal disease | 9,579 (36.8) | 10,740 (27.9) | 0.19 | 31.6 | 31.6 | 0.00 |
|  | Cancer | 2,030 (7.8) | 3,242 (8.4) | 0.02 | 8.3 | 8.2 | 0.00 |
| Immunocompromising condition | | 4,786 (18.4) | 7,620 (19.8) | 0.04 | 19.4 | 19.3 | 0.00 |
| Hospital ward on admission | General ward | 21,569 (82.9) | 31,220 (81.2) | 0.05 | 81.8 | 81.8 | 0.00 |
|  | ICU/step down unit | 4,434 (17.1) | 7,237 (18.8) |  | 18.2 | 18.2 |  |
| Key diagnosis on admission | Sepsis | 138 (0.5) | 139 (0.4) | 0.03 | 0.4 | 0.5 | 0.00 |
|  | Pneumonia | 2,023 (7.8) | 2,803 (7.3) | 0.02 | 7.4 | 7.5 | 0.00 |
| Other COVID-19 treatments at baseline | Anticoagulants | 19,089 (73.4) | 30,148 (78.4) | 0.12 | 76.5 | 76.4 | 0.00 |
|  | Convalescent plasma | 1 (0.0) | 21 (0.1) | 0.38 | 0.0 | 0.0 | 0.00 |
|  | Corticosteroids | 20,400 (78.5) | 35,264 (91.7) | 0.03 | 86.5 | 86.5 | 0.00 |
|  | Baricitinib | 1,356 (5.2) | 1,505 (3.9) | 0.04 | 4.5 | 4.5 | 0.00 |
|  | Tocilizumab | 476 (1.8) | 915 (2.4) | 0.06 | 2.2 | 2.2 | 0.00 |
|  | Oral antivirals | 590 (2.3) | 106 (0.3) | 0.18 | 1.0 | 1.1 | 0.00 |
| Baseline supplemental oxygen requirements | NSOc | 10,269 (39.5) | 12,735 (33.1) | 0.18 | 35.6 | 35.5 | 0.00 |
|  | LFO | 9,191 (35.3) | 14,094 (36.6) |  | 35.9 | 36.0 |  |
|  | HFO/NIV | 5,452 (21.0) | 10,479 (27.2) |  | 25.0 | 24.9 |  |
|  | IMV | 1,091 (4.2) | 1,149 (3.0) |  | 3.6 | 3.6 |  |
| Omicron period | Early (Dec 2021-Dec 2022) | 15,794 (60.7) | 22,281 (57.9) | 0.06 | 59.0 | 59.0 | 0.00 |
|  | Later (Jan 2023-Dec 2024) | 10,209 (39.3) | 16,176 (42.1) |  | 41.0 | 41.0 |  |
| Data is presented as n (%) before matching and as % after matching, unless otherwise indicated.  Abbreviations: COPD, chronic obstructive pulmonary disease; COVID-19, coronavirus disease 2019; HFO/NIV, high-flow oxygen/non-invasive ventilation; ICF, intermediate care facility; ICU, intensive care unit; IMV, invasive mechanical ventilation; IPTW, inverse probability of treatment weighting; LFO, low-flow oxygen; NSOc, no supplemental oxygen charges; SMD, standardized mean difference; SNF, skilled nursing facility | | | | | | | |

**eTable 19. Unadjusted All-cause Inpatient Mortality Rates (PS matching without replacement, COPD Population)**

|  | **Early Omicron (Dec 2021 - Dec 2022)** | | **Later Omicron (Jan 2023- Dec 2024)** | | **Overall (Dec 2021- Dec 2024)** | |
| --- | --- | --- | --- | --- | --- | --- |
|  | **No Remdesivir during the hospitalization** | **Remdesivir in the first two days** | **No Remdesivir during the hospitalization** | **Remdesivir in the first two days** | **No Remdesivir during the hospitalization** | **Remdesivir in the first two days** |
| **Overall Omicron** | **n=12678** | **n=12678** | **n=7730** | **n=7730** | **n=20408** | **n=20408** |
| 14-day mortality | 1473 (11.6) | 1099 (8.7) | 542 (7.0) | 448 (5.8) | 2015 (9.9) | 1547 (7.6) |
| 28-day mortality | 1879 (14.8) | 1434 (11.3) | 642 (8.3) | 546 (7.1) | 2521 (12.4) | 1980 (9.7) |
| **NSOc** | **n=4637** | **n=4637** | **n=2947** | **n=2947** | **n=7584** | **n=7584** |
| 14-day mortality | 314 (6.8) | 259 (5.6) | 148 (5.0) | 111 (3.8) | 462 (6.1) | 370 (4.9) |
| 28-day mortality | 393 (8.5) | 344 (7.4) | 169 (5.7) | 131 (4.4) | 562 (7.4) | 475 (6.3) |
| **Any supplemental oxygen** | **n=8041** | **n=8041** | **n=4783** | **n=4783** | **n=12824** | **n=12824** |
| 14-day mortality | 1159 (14.4) | 840 (10.4) | 394 (8.2) | 337 (7.0) | 1553 (12.1) | 1177 (9.2) |
| 28-day mortality | 1486 (18.5) | 1090 (13.6) | 473 (9.9) | 415 (8.7) | 1959 (15.3) | 1505 (11.7) |

Data is presented as n (%), unless otherwise indicated.

Abbreviations: PS, propensity score; NSOc, no supplemental oxygen charges.

**eTable 20. 14- and 28-day Mortality in Patients Hospitalized for COVID-19 Treated with Remdesivir Within the First 2 Days of Hospitalization vs Those Not Treated with Remdesivir During Hospitalization, by Maximal Supplemental Oxygen Requirements (IPTW, COPD population)**

|  | **aHR [95% CI]** | ***P* value** |
| --- | --- | --- |
| **14-day mortality** |  |  |
| Overall Omicron | 0.74 [0.70-0.79] | < 0.0001 |
| NSOc | 0.76 [0.67-0.86] | < 0.0001 |
| Any supplemental oxygen | 0.75 [0.69-0.80] | < 0.0001 |
| **28-day mortality** |  |  |
| Overall Omicron | 0.76 [0.71-0.80] | < 0.0001 |
| NSOc | 0.79 [0.71-0.88] | < 0.0001 |
| Any supplemental oxygen | 0.75 [0.70-0.80] | < 0.0001 |

Cox proportional hazards model was used to derive estimates adjusted for admission month and time-varying treatment with other COVID-19 medications (baricitinib, tocilizumab, oral antivirals).

Abbreviations: aHR, adjusted hazard ratio; CI, confidence interval; COVID-19, coronavirus disease 2019; IPTW, inverse probability of treatment weighting; NSOc, no supplemental oxygen charges.

**eTable 21. 14- and 28-day Mortality in Patients Hospitalized for COVID-19 Treated with Remdesivir Within the First 2 Days of Hospitalization vs Those Not Treated with Remdesivir Within the First 2 Days, by Maximal Supplemental Oxygen Requirements (PS matching, COPD Population)**

|  | **N** | **aHR [95% CI]** | ***P* value** |
| --- | --- | --- | --- |
| **14-day mortality** |  |  |  |
| Overall Omicron | 44,540 | 0.72 [0.67-0.77] | < 0.0001 |
| NSOc | 16,502 | 0.74 [0.65-0.85] | < 0.0001 |
| Any supplemental oxygen | 28,038 | 0.71 [0.66-0.77] | < 0.0001 |
| **28-day mortality** |  |  |  |
| Overall Omicron | 44,540 | 0.73 [0.69-0.78] | < 0.0001 |
| NSOc | 16,502 | 0.76 [0.68-0.86] | < 0.0001 |
| Any supplemental oxygen | 28,038 | 0.72 [0.67-0.77] | < 0.0001 |

Cox proportional hazards model was used to derive estimates adjusted for admission month and time-varying treatment with other COVID-19 medications (baricitinib, tocilizumab, oral antivirals).

Abbreviations: aHR, adjusted hazard ratio; CI, confidence interval; COVID-19, coronavirus disease 2019; NSOc, no supplemental oxygen charges; PS, propensity score.

**eFigure 1. Balance of absolute standardized mean difference before and after PS matching among adults hospitalized for COVID-19 (overall population)**


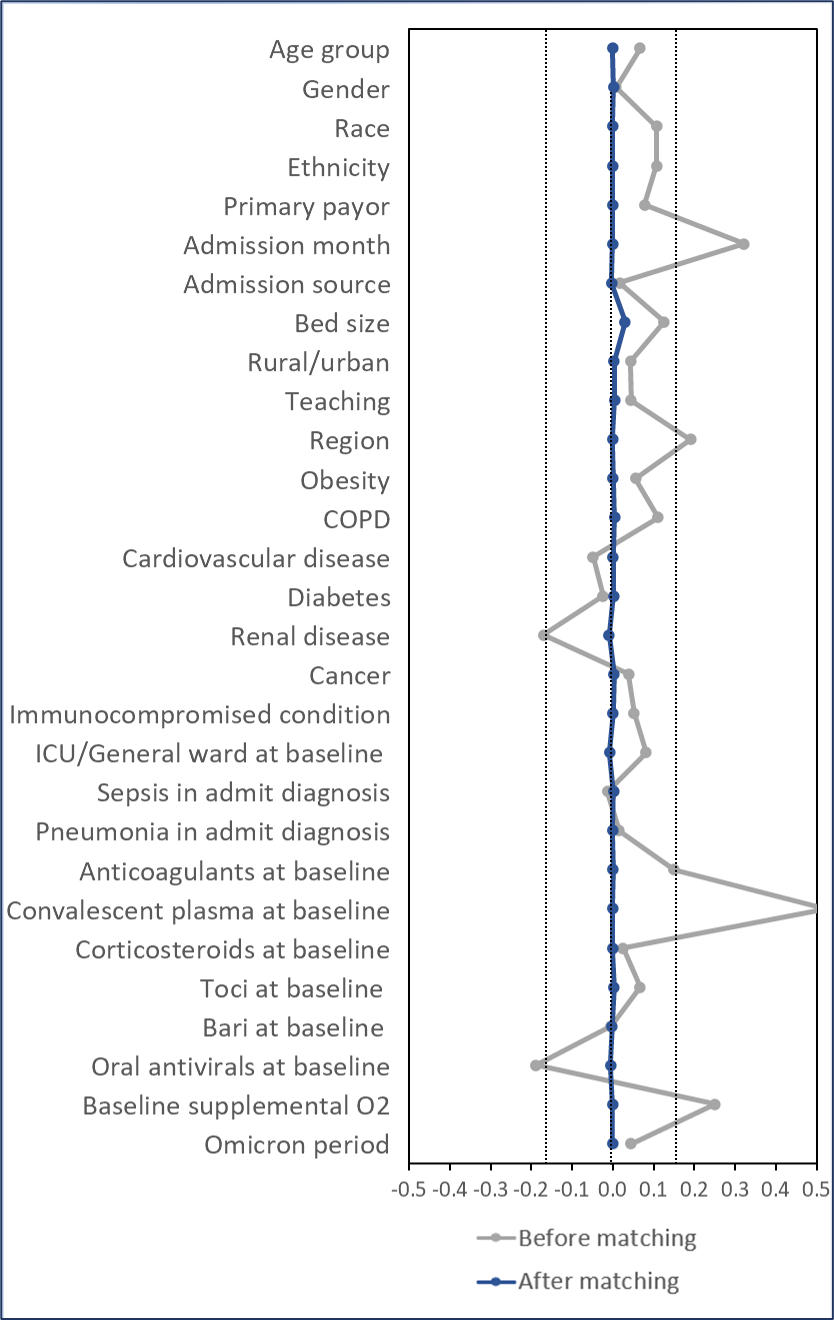


Abbreviations: COPD, Chronic Obstructive Pulmonary Disease; ICU, intensive care unit; O2, oxygen; PS, propensity score.

**eFigure 2. Balance of absolute standardized mean difference before and after PS matching among patients hospitalized for COVID-19 (elderly population)**


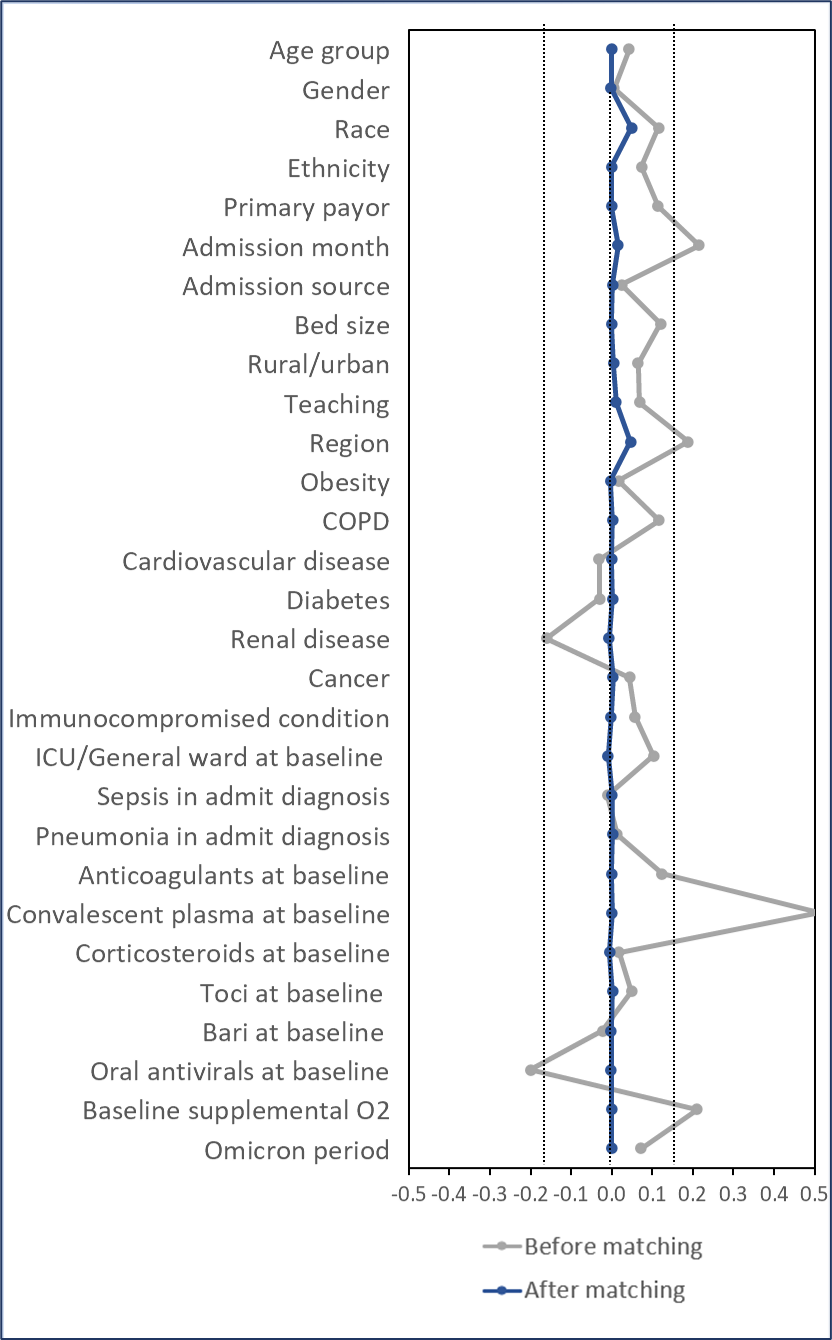


Abbreviations: COPD, Chronic Obstructive Pulmonary Disease; ICU, intensive care unit; O2, oxygen; PS, propensity score.

**eFigure 3. Balance of absolute standardized mean difference before and after PS matching among patients hospitalized for COVID-19 (pneumonia population)**


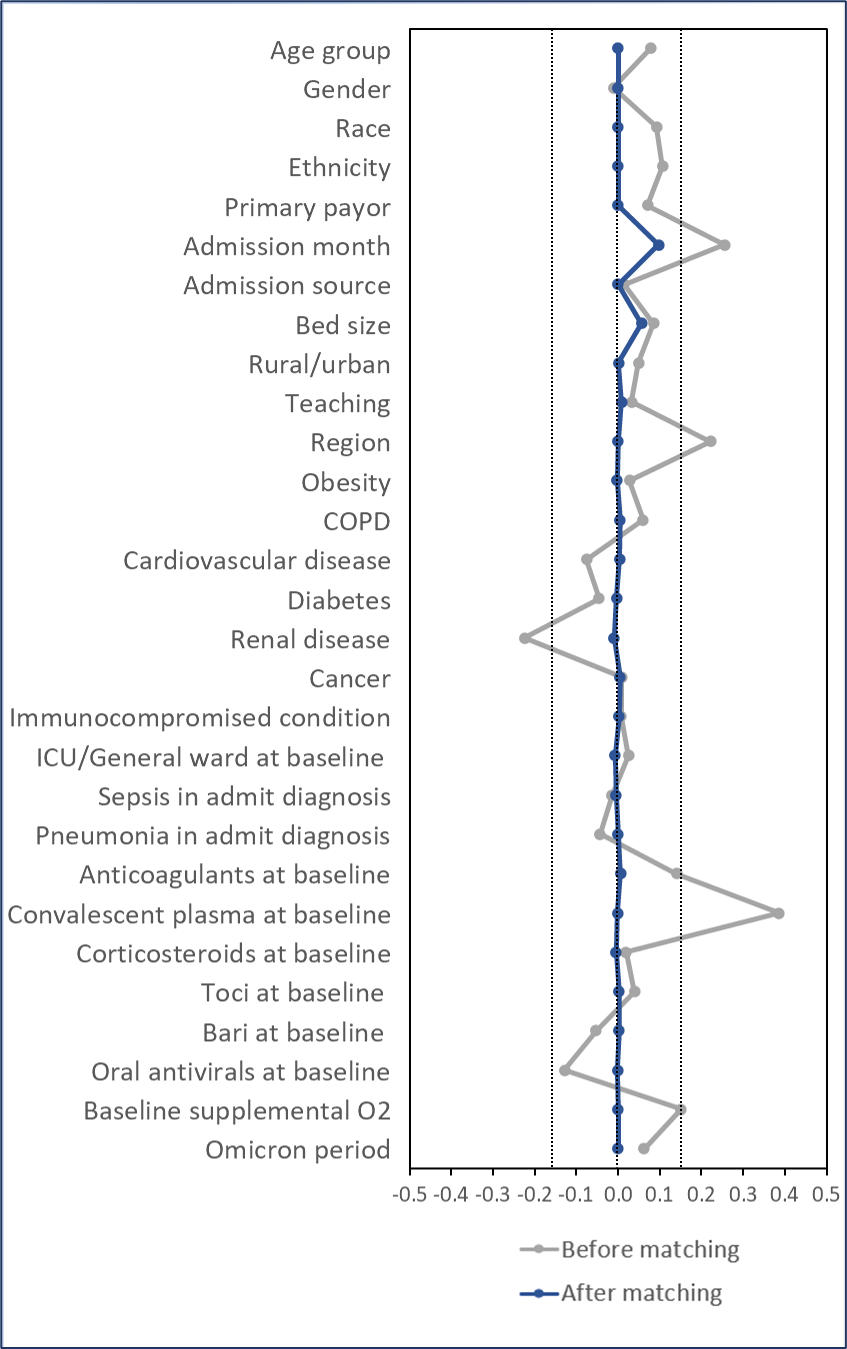


Abbreviations: COPD, Chronic Obstructive Pulmonary Disease; ICU, intensive care unit; O2, oxygen; PS, propensity score.

**eFigure 4. Balance of absolute standardized mean difference before and after PS matching among patients hospitalized for COVID-19 (COPD population)**


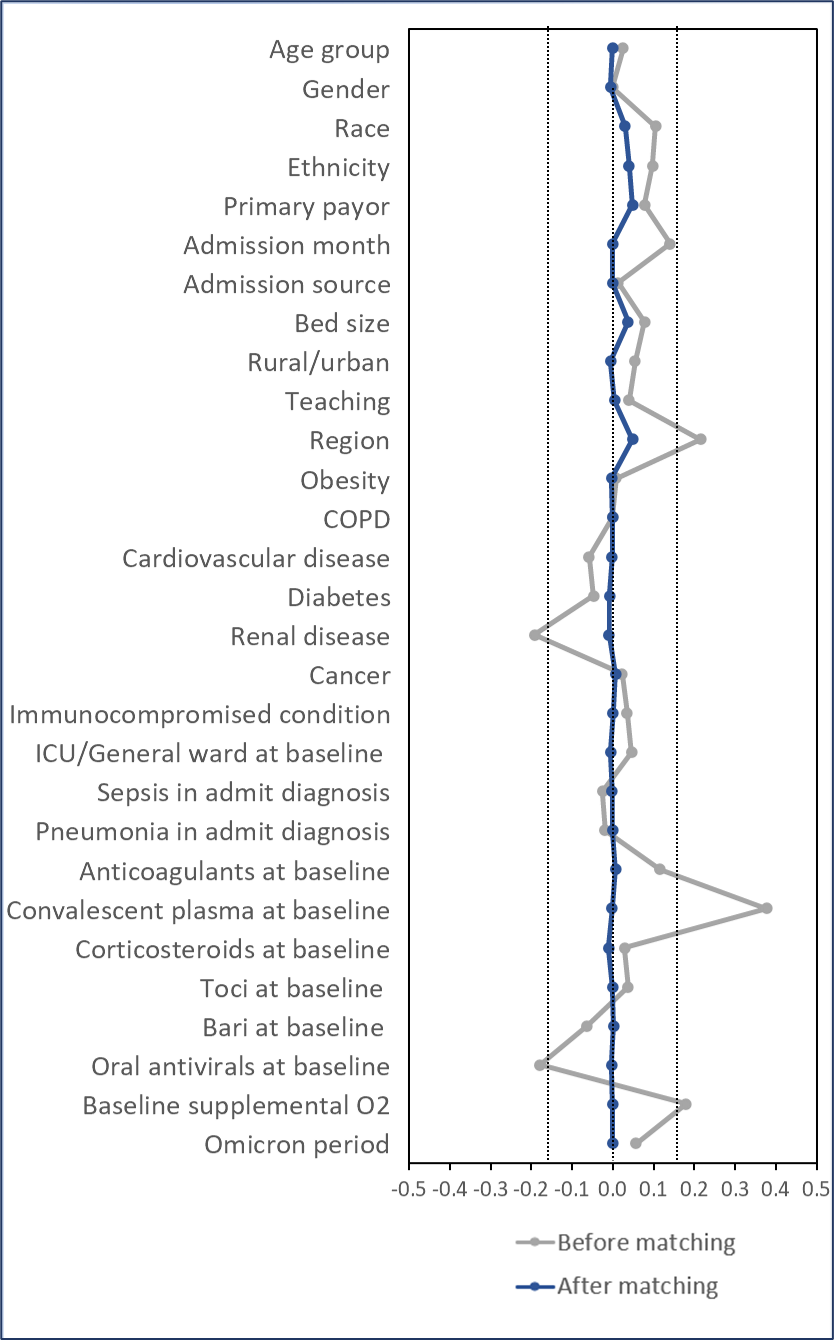


Abbreviations: COPD, Chronic Obstructive Pulmonary Disease; ICU, intensive care unit; O2, oxygen; PS, propensity score.
